# SAFA facilitates chromatin opening of immune genes through interacting with anti-viral host RNAs

**DOI:** 10.1371/journal.ppat.1010599

**Published:** 2022-06-03

**Authors:** Lili Cao, Yujie Luo, Xuefei Guo, Shengde Liu, Siji Li, Junhong Li, Zeming Zhang, Yingchi Zhao, Qiao Zhang, Feng Gao, Xiong Ji, Xiang Gao, Yunfei Li, Fuping You

**Affiliations:** 1 Department of Immunology, School of Basic Medical Sciences, Beijing Key Laboratory of Tumor Systems Biology, Institute of Systems Biomedicine, Peking University Health Science Center, Beijing, China; 2 Beijing Institutes of Life Science, Chinese Academy of Sciences, Beijing, China; 3 Department of Gastrointestinal Oncology, Key Laboratory of Carcinogenesis and Translational Research (Ministry of Education/Beijing), Peking University Cancer Hospital and Institute, Beijing, China; 4 CAS Key Laboratory of Infection and Immunity, National Laboratory of Macromolecules, Institute of Biophysics, University of Chinese Academy of Sciences, Chinese Academy of Sciences, Beijing, China; 5 School of Medicine, Jinan University, Guangzhou, Guangdong, China; 6 Key Laboratory of Cell Proliferation and Differentiation of the Ministry of Education, School of Life Sciences, Peking-Tsinghua Center for Life Sciences, Peking University, Beijing, China; 7 State Key Laboratory of Microbial Technology, Microbial Technology Institute, School of life science, Shandong University, Qingdao, China; Albany Medical College, UNITED STATES

## Abstract

Regulation of chromatin structure and accessibility determines the transcription activities of genes, which endows the host with function-specific patterns of gene expression. Upon viral infection, the innate immune responses provide the first line of defense, allowing rapid production of variegated antiviral cytokines. Knowledge on how chromatin accessibility is regulated during host defense against viral infection remains limited. Our previous work found that the nuclear matrix protein SAFA surveilled viral RNA and regulated antiviral immune genes expression. However, how SAFA regulates the specific induction of antiviral immune genes remains unknown. Here, through integration of RNA-seq, ATAC-seq and ChIP-seq assays, we found that the depletion of SAFA specifically decreased the chromatin accessibility, activation and expression of virus induced genes. And mutation assays suggested that the RNA-binding ability of SAFA was essential for its function in regulating antiviral chromatin accessibility. RIP-seq results showed that SAFA exclusively bound with antiviral related RNAs following viral infection. Further, we combined the CRISPR-Cas13d mediated RNA knockdown system with ATAC-qPCR, and demonstrated that the binding between SAFA and according antiviral RNAs specifically mediated the openness of the corresponding chromatin and following robust transcription of antiviral genes. Moreover, knockdown of these associated RNAs dampened the accessibility of related genes in an extranuclear signaling pathway dependent manner. Interestingly, VSV infection cleaved SAFA protein at the C-terminus which deprived its RNA binding ability for immune evasion. Thus, our results demonstrated that SAFA and the interacting RNA products collaborated and remodeled chromatin accessibility to facilitate antiviral innate immune responses.

## Author summary

Regulation of chromatin opening and gene expression underlies a key point during host defense against viral infection, which endows the host with timely and effective antiviral gene expression patterns. We previously reported that the nuclear matrix protein SAFA surveils viral RNA and regulates antiviral immune genes expression. However, how SAFA regulates the expression and what determines the specific induction of antiviral immune genes remains unclear. Here, we used a combination of high-throughput sequencing technologies and found that SAFA and the interacting RNA products collaborated and specifically remodeled chromatin accessibility to facilitate antiviral immune genes expression. We also found that VSV infection cleaved SAFA protein at the C-terminus and deprived its RNA binding ability for immune evasion. Our study provides new insights into the mechanism by which chromatin remodeling facilitates the induction of antiviral immune genes.

## Introduction

In the eukaryotic cell nucleus, the chromatin structures are hierarchical ordered at different levels, ranging from kilobase to megabase scales [1, 2]. The multiple levels including nucleosome, loops, topologically associated domains (TADs), A/B compartments and territories [3–5]. Chromatin is the template of all DNA-related processes. The proper regulation of chromatin structure and the subsequent accessibility of DNA are essential for the performance of numerous cellular functions [6–10]. Upon viral infection, the innate immune responses provide the first line of defense, allowing rapid production of variegated anti-viral cytokines [11–13]. This process is primarily controlled by dynamic organization of the genome, which reprogrammed the specific genomic regions from a condensed state to a transcriptionally accessible state [14, 15]. Hence, there should be a precise molecular mechanism underpinning the remodeling of chromatin during host defense against virus infection.

Processes involved in the alteration of chromatin accessibility are diverse, including post-translational modifications of histones, incorporation of histone variants, DNA methylation and ATP-dependent chromatin remodeling [16–19]. There is accumulating evidence indicating that RNAs also play an important role in regulating chromatin accessibility [20–24]. The RNA encoded by HOXC locus represses transcription of the HOXD locus through interacting with the polycomb repressive complex 2 (PRC2) [25]. During the process of mammal X-chromosome inactivation, the stable repression of all X-linked genes is mediated by the long noncoding RNA, Xist, which is transcribed from specific X-linked sequences [26]. Xist induces a cascade of chromatin changes, including post-translational histone modifications and DNA methylation, by interacting with multiple proteins. 62%–75% of the human genome is capable of producing various RNA species, but less than 2% encodes proteins [27]. RNAs reflect the direct production of the genetic information encoded by genomes. In addition, RNAs production is highly dynamic that different species and amounts of RNAs are produced at different stages of transcription [28, 29]. These led us to wonder whether the openness of chromatin are regulated by the RNA products during viral infection.

Scaffold attachment factor A (SAF-A), also known as heterogeneous ribonucleoprotein U (HNRNP-U), is an abundant nuclear matrix associated protein [30]. Canonically, SAFA is an RNA-binding protein mainly involved in regulating gene transcription and RNA splicing [31]. Several reports suggested that SAFA played a critical role in the recruitment of Xist RNA in inactive X chromosome [32]. Recently, SAFA was demonstrated to play a central role in regulating chromatin architecture. The *in situ* Hi-C assay showed that SAFA mainly binds to active chromatin [33]. Disruption of SAFA led to compartment switching from B to A and reduced the TAD boundary strengths at borders between two types of compartments [33]. Nozawa et al. reported that oligomerized SAFA remodeled interphase chromatin structures through interacting with nascent RNAs [34]. SAFA oligomerization decompacted large-scale chromatin structure while SAFA deficiency or monomerization promoted aberrant chromosome folding [34]. Our previous study suggested that SAFA surveilled viral RNA in the nucleus and facilitated innate immune response by activating antiviral enhancers and super-enhancers [35]. Interestingly, this process was also dependent on SAFA oligomerization. Viral infection induced SAFA oligomerization, which was essential for the activation of antiviral immune responses [35]. However, it is unknown if or how SAFA regulates the accessibility of the specific chromatin locus coding antiviral genes during virus infection. Moreover, we previously found that SAFA was also able to interact with host RNA during virus infection (35). The function of SAFA-host RNA binding remains elusive.

In the present study, by combining Assay for Transposase-Accessible Chromatin with high throughput sequencing (ATAC-seq), Chromatin immunoprecipitation followed by sequencing (ChIP-seq) and bulk RNA- sequencing (RNA-seq), we assessed the genome-wide chromatin accessibility and gene expression in wild type and SAFA deficient cells after viral infection, and found that SAFA was essential for increasing the chromatin accessibility and activating the induction of antiviral immune genes. In addition, this process is dependent on the association of SAFA with anti-viral transcripts. Mechanistically, RNAs produced during viral infection interacted with SAFA and facilitated the remodeling of related chromatin regions in an extranuclear antiviral signaling pathways dependent manner. Intriguingly, on the other hand, viral infection induced cleavage of SAFA and dampened its RNA binding activity for immune evasion. Hence, the canonical antiviral pathways directed the production of antiviral transcripts, which bound to and activated SAFA, and in turn SAFA further facilitated transcription of antiviral genes by increasing the accessibility of chromatin.

## Results

### SAFA deficiency decreased the chromatin accessibility of antiviral immune genes

**Fig 1:**
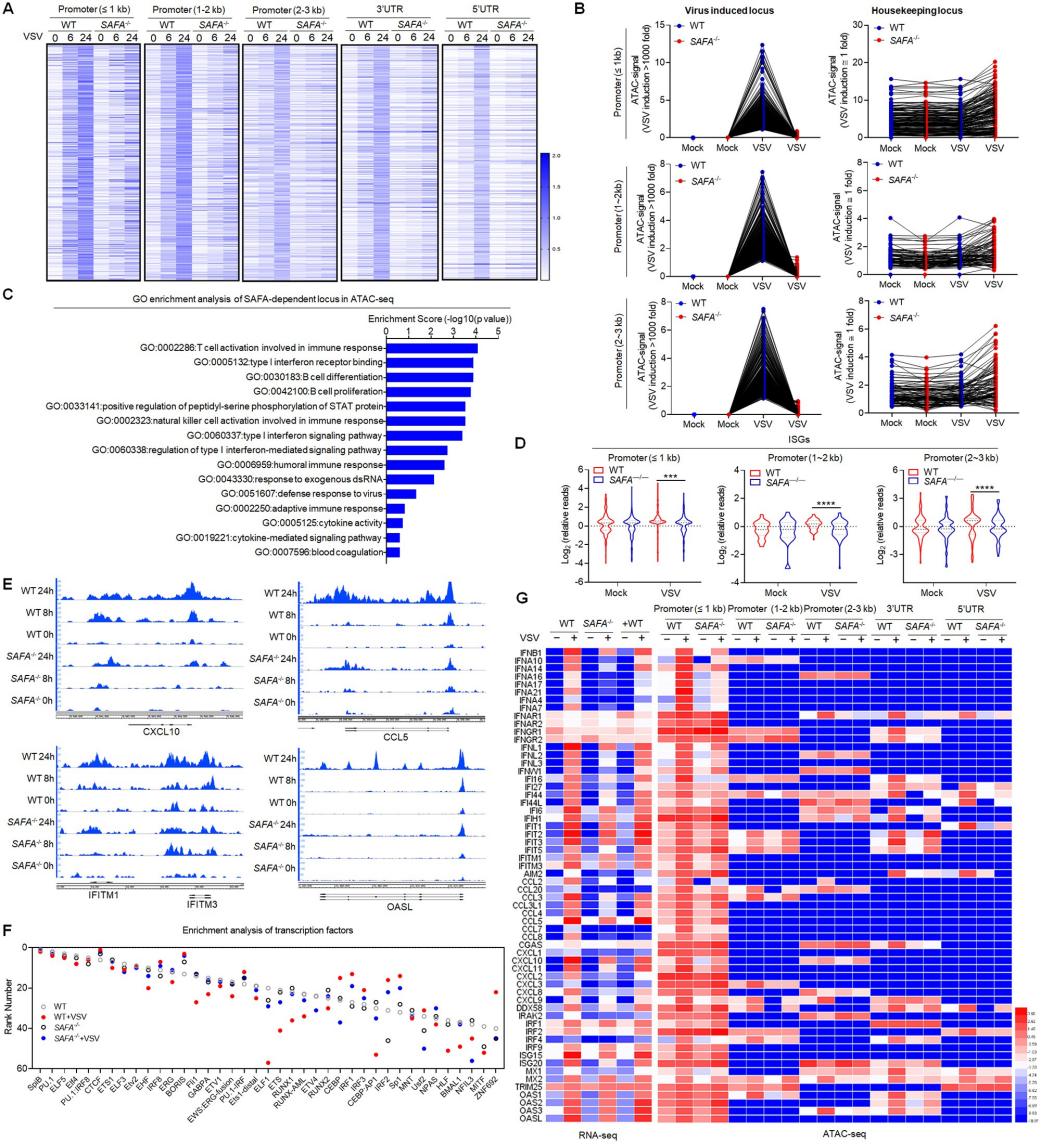
SAFA deficiency decreased the chromatin accessibility of antiviral immune genes. (A) Heatmap (log RPKM+1) showing the ATAC-seq signal in Wild-type (WT) and *SAFA*^−/−^ THP-1 cells with VSV infection for indicated hours. (B) Line graph (RPKM) showing SAFA in regulation of VSV-induced accessible locus and housekeeping locus in ATAC-seq. (C) GO term enrichment analysis of genes significantly affected by SAFA depletion in ATAC-seq. (D) Violin graph (log RPKM) showing ISGs affected by SAFA depletion in ATAC-seq. (E) Genome browser views of ATAC-seq signal for the indicated genes. (F) Transcription factor enrichment analysis of ATAC-seq. (G) Heatmap comparing ATAC-seq signal (log RPKM+1) and RNA-seq signal (log FPKM+1) of indicated genes. ***p < 0.001, ****p < 0.0001 (Student’s t test; D). The cells were infected by VSV at 0.1 multiplicity of infection (MOI). Data were pooled from two independent experiments (A-D, F and G). Data were representative of two independent experiments (E).

Vesicular stomatitis virus (VSV), a single-stranded negative sense RNA virus, causes vesicular stomatitis with a broad host range from insects to mammals. Our previous study found that SAFA deficiency showed a more visible impact on the innate immune response induced by VSV infection compared with Herpes simplex virus type 1 (HSV-1) infection (35). To explore the potential role of SAFA in regulating chromatin accessibility during viral infection, we performed ATAC-seq and RNA-seq analysis in wild type and SAFA deficient (S*AFA*^−/−^) THP-1 cells with or without VSV infection (S1A Fig). SAFA deficiency led to an extensive decrease in chromatin accessibility at both the promoter and the UTR regions, particularly at 24 hours after VSV infection (Fig 1A and S1B Fig). Intriguingly, the decrease of chromatin accessibility by SAFA disruption exclusively took place at the locus governing the expression of viral induced genes, but not housekeeping locus (Fig 1B and S1C Fig). The openness of the locus was induced over 1000-fold in THP-1 cells after VSV infection for 24 hours, which was apparently impaired due to SAFA depletion. While the loci where the accessibility was not induced by viral infection showed no significant differences between wild-type and S*AFA*^−/−^ cells before or after virus infection (Fig 1B and S1C Fig). The Gene Ontology (GO) term enrichment analysis showed that these genes significantly affected by SAFA depletion were involved in type I interferon signaling pathway and host defense response to virus (Fig 1C). Type I interferons (IFNs) and IFN-stimulated genes (ISGs) are potent innate anti-viral immune response effectors. IFNs signaling induces the expression of a large number of genes, collectively referred to as ISGs that generally function to inhibit viral replication (36). Consistently, the chromatin accessibility of related ISGs were greatly decreased in SAFA deficient cells, but not housekeeping genes (Fig 1D and 1E, S1D and S1E Fig). *CXCL10*, *CCL5*, *IFITM1/3* and *OASL* are known to code important antiviral effectors. *CXCL10*, *IFITM1/3* and *OASL* are important ISGs [36, 37]. CCL5 is a T cell chemoattractant that is critical for immune control of viral infections [38]. The virus induced chromatin accessibility of these genes was robustly decreased in *SAFA*^−/−^ cells compared with wild-type cells (Fig 1E). The transcription factor enrichment analysis revealed a loss of accessibility for genes regulated by IRF3, IRF1, IRF8 and IRF2 (Fig 1F). Notably, interferon regulatory factors (IRF) target genes have a critical role in the regulation of host defense [39]. IRFs are best known for their critical role as transcription factors promoting Type I IFN expression. *ISG15* is one of the typical ISGs regulated by IRF in the antiviral process. Consistently, ATAC-seq results showed that the accessibility of *IFNβ* and *ISG15* were obviously reduced in *SAFA*^-/-^ cells compared with wild-type cells (S1E Fig). In addition, we validated these results with ATAC-qPCR, which showed similar results (S1F Fig).

Integrated analysis of RNA-seq and ATAC-seq revealed that genes with reduced chromatin accessibility also showed significant lower expression levels (Fig 1G). Correspondingly, the downregulated genes in SAFA deficient cells after VSV infection were mainly enriched in innate immune response to virus (S1F Fig). Moreover, we measured the cell viability after VSV infection at different time points. Compared with non-infected cells, we observed a comparable cell viability of cells infected by 0.1 MOI VSV (S1G Fig). Together, these results suggested that SAFA mediated the chromatin accessibility of antiviral immune genes after viral infection.

### SAFA deficiency decreased the induction of antiviral immune genes

**Fig 2:**
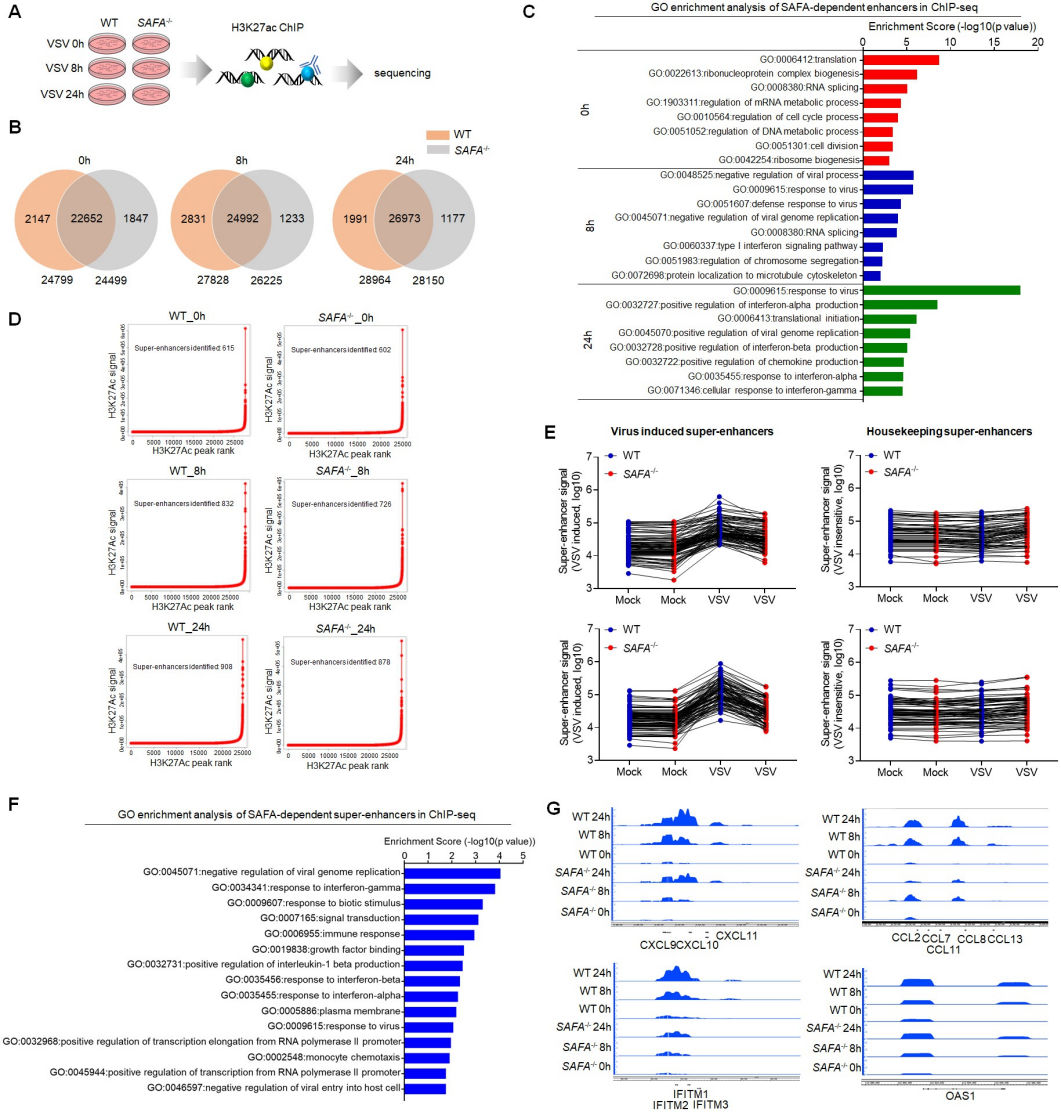
SAFA deficiency decreased the activation of antiviral immune genes. (A) Models depicting the ChIP-seq assay of H3K27ac in Wild-type (WT) and *SAFA*^−/−^ THP-1 cells with VSV infection for indicated hours. (B) Venn diagram showing amounts of enhancers in WT and *SAFA*^−/−^ THP-1 cells with VSV infection. (C) GO term enrichment analysis of enhancers-related genes affected by SAFA depletion in ChIP -seq. (D) Delineation of -enhancers super based on H3K27Ac occupancy in WT and *SAFA*^−/−^ THP-1 cells with VSV infection using the ROSE algorithm. (E) Line graph (RPKM) showing SAFA in regulation of VSV-induced and housekeeping supper-enhancer formation. (F) GO term enrichment analysis of super-enhancers related genes affected by SAFA depletion in ChIP -seq. (G) Genome browser views of ChIP -seq signal for the indicated genes. The cells were infected by VSV at 0.1 MOI. Data were pooled from two independent experiments (B-F). Data were representative of two independent experiments (G).

Enhancers and promoters are key regulatory DNA elements that control the expression of genes [40, 41]. The accessible chromatin reflects a permission for physical interactions of transcription machineries with enhancers and promoters [14]. To confirm the role of SAFA in enhancing the chromatin accessibility of antiviral genes, we performed ChIP-seq analysis with H3 lysine 27 acetylation (H3K27ac) antibody. Enhancer activation was marked by high level of H3K27ac [42]. Our previous results showed that SAFA facilitates distal enhancer activation of type I IFN [35]. Here we assessed the impact of SAFA on activation of virus-induced enhancers in a genome-wide scale (Fig 2A and S2A Fig). SAFA deficiency downregulated the activation of enhancers globally after VSV infection. There were 24799 enhancers in resting wild type THP-1 cells, 27828 after VSV infection for 8 hours, and 28964 after VSV infection for 24 hours. As for *SAFA*^−/−^ cells, there were 24499 enhancers in resting cells, 26225 after VSV infection for 8 hours, and 28150 after VSV infection for 24 hours (Fig 2B). The difference can be even greater at the early stage of infection (S2B Fig). Moreover, these enhancers inactivated by SAFA disruption were mainly involved in antiviral immune responses (Fig 2C).

Super-enhancers are clusters of enhancers across a long range of genomic DNA, which drive expression of genes that define cell state [43, 44]. It was also marked by H3K27ac. Further analysis showed that SAFA is required for the activation of super-enhancers induced by viral infection. There were 615 super-enhancers in resting THP-1 cells, 832 after VSV infection for 8 hours, and 908 after VSV infection for 24 hours. In *SAFA*^−/−^ cells, there were 602 super-enhancers in untreated cells, 726 after VSV infection for 8 hours, and 878 after VSV infection for 24 hours (Fig 2D). SAFA deficiency decreased the formation of super-enhancers after VSV infection, especially at the early stage of infection (Fig 2E and S2C Fig). Meanwhile, SAFA depletion showed no obvious impact on the formation of super-enhancers that insensitive to VSV infection (Fig 2E), suggesting that SAFA mainly affected the activation of super-enhancers related to viral infection. The enrichment analysis suggested that these super-enhancers associated genes were involved in immune responses and host defense to virus, which were significantly downregulated by SAFA depletion (Fig 2F). The formation of super-enhancers governing *CXCL9/10/11*, *CCL7/8/13*, *IFITM1/2/3*, *OAS1* genes, but not housekeeping genes, was robustly decreased in SAFA mutation cells after virus infection (Fig 2G and S2D Fig). And ChIP-qPCR showed similar results (S2E Fig). In eukaryotes, RNA polymerase II (pol II) transcribes all protein-coding genes and most non-coding RNA genes. Using ChIP against RNA pol II can simply mapped the transcription level of actively transcribing genes [45]. To further validate the results, we did ChIP-qPCR with RNA pol II antibody in wild-type and *SAFA*^−/−^ cells after VSV infection. Consistently, SAFA deficiency decreased the occupancy of RNA pol II at these ISGs (S2F Fig). Moreover, the majority of these impaired super-enhancer-driven genes were protein-coding genes, but there was also a considerable part of non-coding genes (S2G Fig). Therefore, SAFA was required for the activation of virus induced enhancers/super-enhancers.

### RNA binding activity of SAFA is critical for increasing the accessibility of anti-viral chromatin

We then investigated the mechanism by which SAFA specifically increased chromatin accessibility of antiviral genes after infection. In the interphase, SAFA remodels the chromatin structures through the interaction with nascent RNAs [34]. An increasing body of evidence suggested that RNAs were involved in regulation of chromatin accessibility. By using genome-wide binding profiling, Kambiz et al. showed that eRNAs regulated genomic accessibility of the transcriptional complex to defined regulatory regions [23]. Dong et al. reported that the lncRNA, *LncMyoD*, regulated lineage determination and progression through modulating chromatin accessibility [22]. Our previous results suggest that SAFA facilitated anti-viral innate immune responses, which was also dependent on the RNA-binding ability [35]. These prompted us to investigate whether the regulation of chromatin accessibility by SAFA during virus infection is also dependent on the interaction with RNAs.

**Fig 3:**
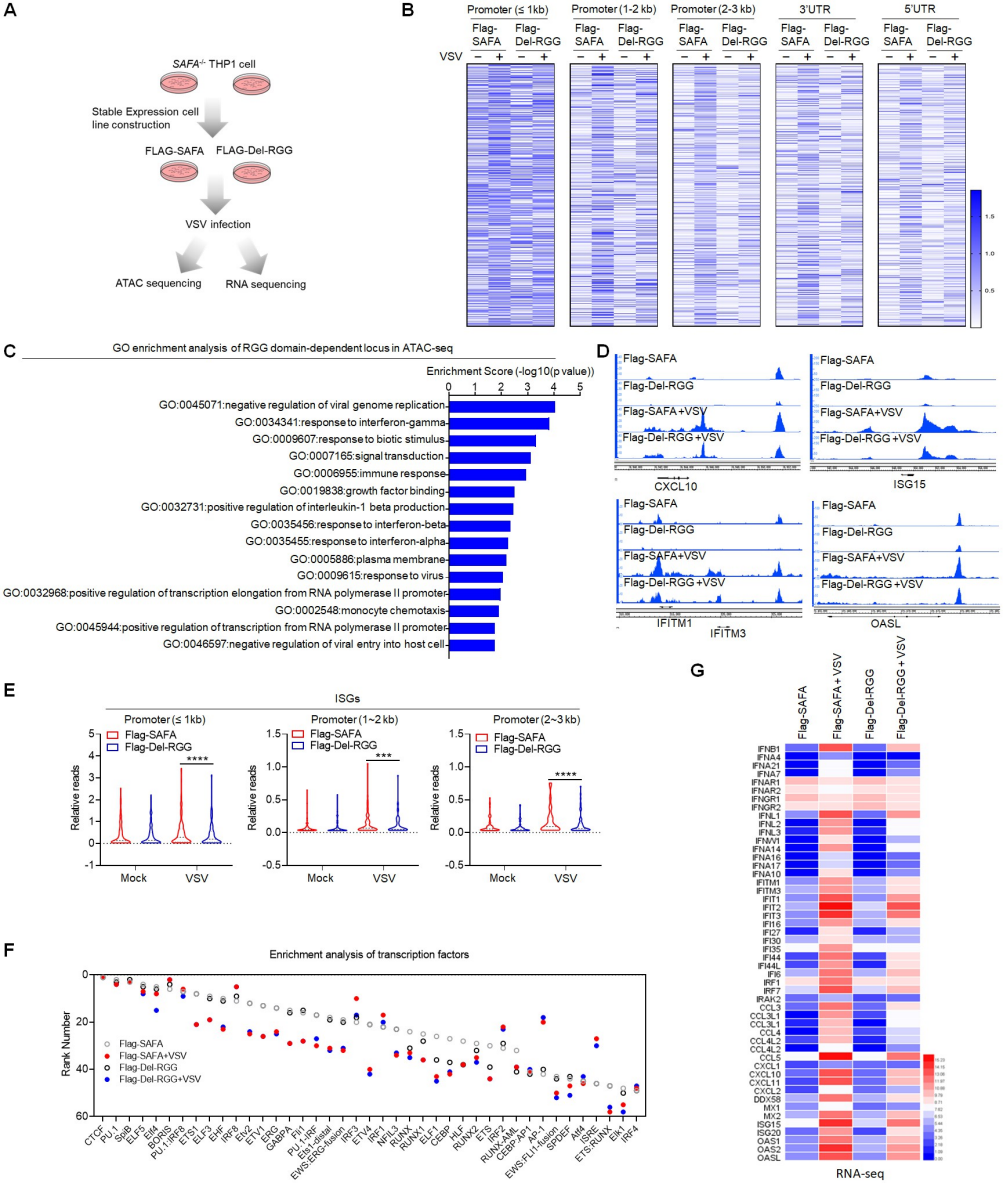
RNA binding activity of SAFA is critical for increasing the accessibility of anti-viral chromatin. (A) Models depicting the ATAC-seq and RNA-seq assay in Flag-SAFA and Flag-Del-RGG stably expressed *SAFA*^−/−^ THP-1 cells with VSV infection for 6 hours. (B) Heatmap (log RPKM+1) showing the ATAC-seq signal. (C) GO term enrichment analysis of genes significantly affected by RGG domain depletion in ATAC-seq. (D) Genome browser views of ATAC-seq signal for the indicated genes. (E) Violin graph (log RPKM) showing ISGs affected by RGG domain depletion in ATAC-seq. (F) Transcription factor enrichment analysis of ATAC-seq. (G) Heatmap (log FPKM+1) showing RNA-seq signal for the indicated genes. ***p < 0.001, ****p < 0.0001 (Student’s t test; E). The cells were infected by VSV at 0.1 MOI. Data were pooled from two independent experiments (B, C and E-G). Data were representative of two independent experiments (D).

Structurally, SAFA contains an N-terminal DNA-binding domain, an ATP-binding AAA+ domain, a SPRY domain and an RNA-binding RGG repeat at the C-terminal [46, 47]. We reconstituted the full-length (Flag-SAFA) and RGG domain depleted (Flag-Del-RGG) Flag tagged SAFA plasmids into *SAFA*^−/−^ THP-1 cells, which enabled their stable expression (S3A Fig). And the native PAGE result suggested that the RGG mutantion abolished the RNA-binding ability of SAFA (S3A Fig) (35). Further, we did ATAC-seq and RNA-seq with both cell lines (Fig 3A). The results showed that after VSV infection, the genome-wide chromatin accessibility was downregulated in RGG domain mutated cells compared with that in Flag-SAFA cells (Fig 3B and S3B Fig). The GO enrichment analysis showed that these reduced genes were mainly involved in immune response and response to interferon (Fig 3C). Consistently, the chromatin accessibility of ISGs were significantly decreased in RGG domain depleted cells, but not housekeeping genes (Fig 3D and 3E and S3C–S3E Fig). The genome-wide transcription factor enrichment analysis inferred an impaired association of genes with IRF motifs in them (Fig 3F).

Moreover, the expression of anti-viral genes was obviously downregulated in Flag-Del-RGG cells (Fig 3G). RGG domain depletion mainly affected the regulation of type I interferon-mediated signaling pathway following viral infection (S3F Fig). These results suggested that the RNA-binding ability was essential for SAFA in increasing chromatin accessibility of antiviral immune genes during viral infection.

### SAFA interacted with antiviral host RNAs in a time-dependent manner during viral infection

**Fig 4:**
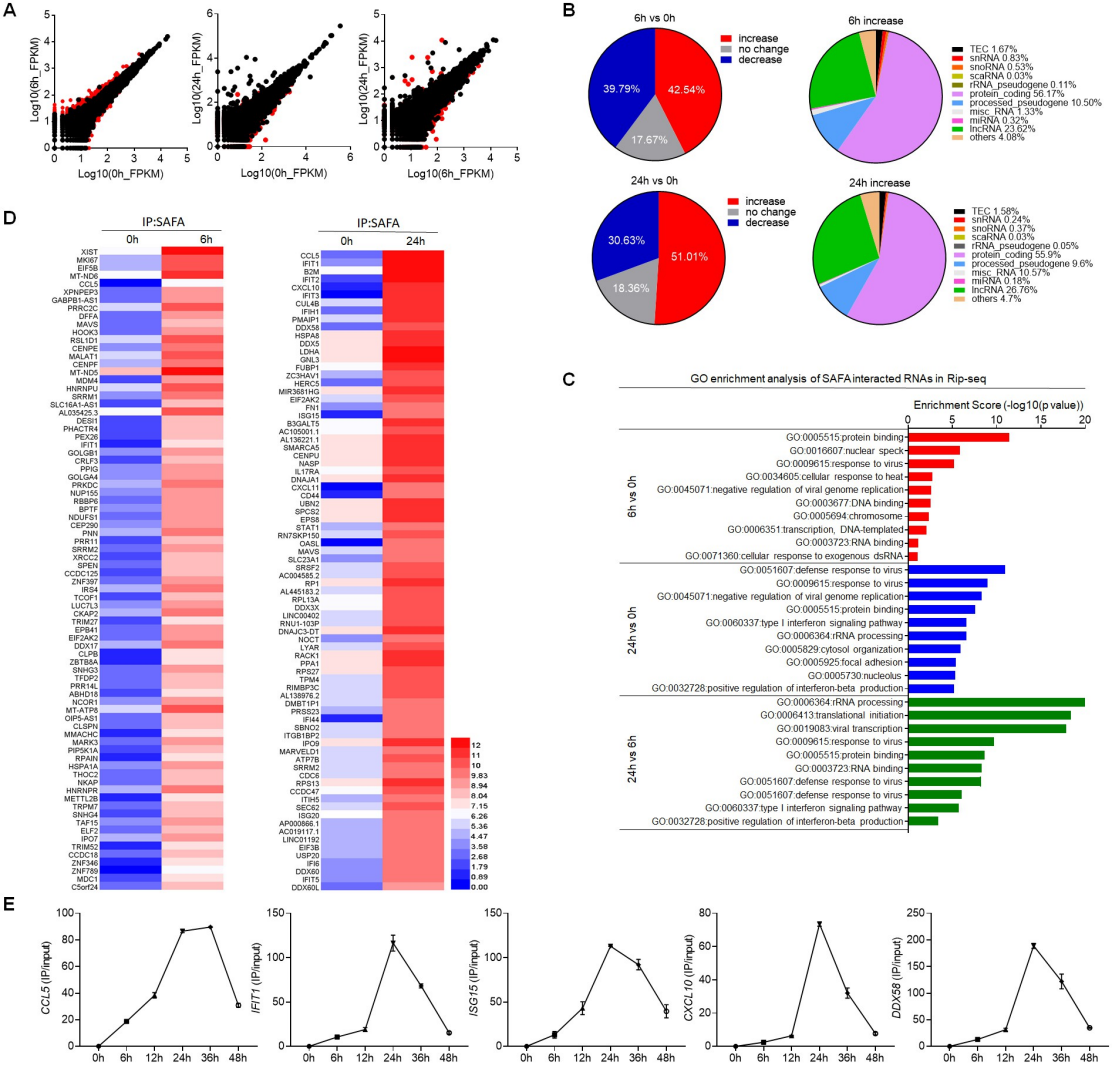
SAFA interacted with antiviral related RNAs in a time-dependent manner during viral infection. (A) Scatter diagram showing differential RNA binding profiles of SAFA in THP-1 cells with VSV infection for 6 hours or 24 hours. (B) Pie chart showing the changes of RNAs interacted with SAFA in RIP-seq upon VSV infection (left); pie chart showing the distribution profile of RNAs with increased interaction with SAFA after VSV infection (right). (C) GO term enrichment analysis of RNAs interacted with SAFA in RIP-seq. (D) Heatmap (log RPKM+1) showing RIP-seq signal for the indicated RNAs. (E) Line graph showing time-dependent RNA binding manner of indicated genes with VSV infection for indicated times. The cells were infected by VSV at 0.1 MOI. Data were pooled from two independent experiments (A-D). Data were pooled from three experiments (E).

In our previous paper, we performed RNA immunoprecipitation sequencing (RIP-seq) analysis with the cells infected by HSV-1 (35). SAFA was able to interact with both viral RNA and host RNA. The robustly increased abundance of viral RNA and double-stranded RNA structure triggers the oligomerization of SAFA (35). However, it is unknown the function of the binding between SAFA and host RNA during virus infection. To gain further insight into the role of RNA-binding ability of SAFA in chromatin structure regulation after viral infection, we performed RIP-seq of THP-1 cells following VSV infection for 6 hours and 24 hours (S4 Fig) [48]. Further we selectively analyzed the immunoprecipitated RNA from host. SAFA showed differential binding profiles at different stages of viral infection (Fig 4A). The interaction between host RNAs and SAFA were increased by 42.54% and 51.01% after VSV infection for 6 hours and 24 hours respectively (Fig 4B). More than 50% of the total increased SAFA binding RNAs were protein coding mRNAs after VSV infection (Fig 4B). There is also a considerable part of noncoding RNAs (ncRNAs), especially lncRNA (Fig 4B).

GO enrichment analysis revealed that these RNAs differentially interacting with SAFA were mainly involved in response to virus and protein binding after viral infection for 6 hours (Fig 4C). At the later stage of infection, these associated RNAs were almost entirely related to defense response to virus (Fig 4C). Among those RNAs, *CCL5*, *IFIT1/2/3*, *CXCL10*, *MAVS*, *DDX58*, *ISG15*, *OASL* and *IFI44* are known to encode important innate antiviral effectors (Fig 4D) [36–38, 49]. Furthermore, the results with more detailed time points suggested that the interaction of SAFA with these RNAs showed a time-dependent manner, in which the binding first increased with the time after VSV infection, peaked at about 24 hours and then dropped around 48 hours (Fig 4E). Further, we performed RIP assay with Flag antibody in HEK293T cells overexpressed with Flag-HNRNP A, Flag-HNRNP R or Flag-SAFA plasmids. RIP-qPCR results suggested that SAFA showed significantly stronger affinity to anti-viral RNAs than the other HNRNPs (S4B Fig). The HNRNPs represent a large family of RNA-binding proteins. This result suggested that the binding of SAFA with antiviral RNAs was specifically induced by viral infection, but not a general phenomenon of RNA binding proteins caused by the increase in the total amount of viral RNA. Thus, these results showed that SAFA interacted with antiviral related RNAs in a time dependent manner after viral infection.

### SAFA-interacting RNA mediated specific chromatin remodeling in an extranuclear pathway dependent manner

There is accumulating evidence suggests that RNA molecules are components of and play regulatory roles at different stages of transcription. Recent studies have shown that RNAs produced during early steps in transcription initiated the transcriptional condensate formation [50]. Moreover, the regulation roles of RNAs in gene expression showed locus-specific characteristics, which tends to regulate the expression of adjacent or related genes [22, 23, 25, 26]. The RNA binding-dependent regulatory activity of SAFA, coupled with evidence that the associated RNAs are mainly antiviral innate immunity related, led us to wonder whether the SAFA-interacting RNA mapped or characterized the regulatory regions of accessible chromatin during viral infection.

**Fig 5:**
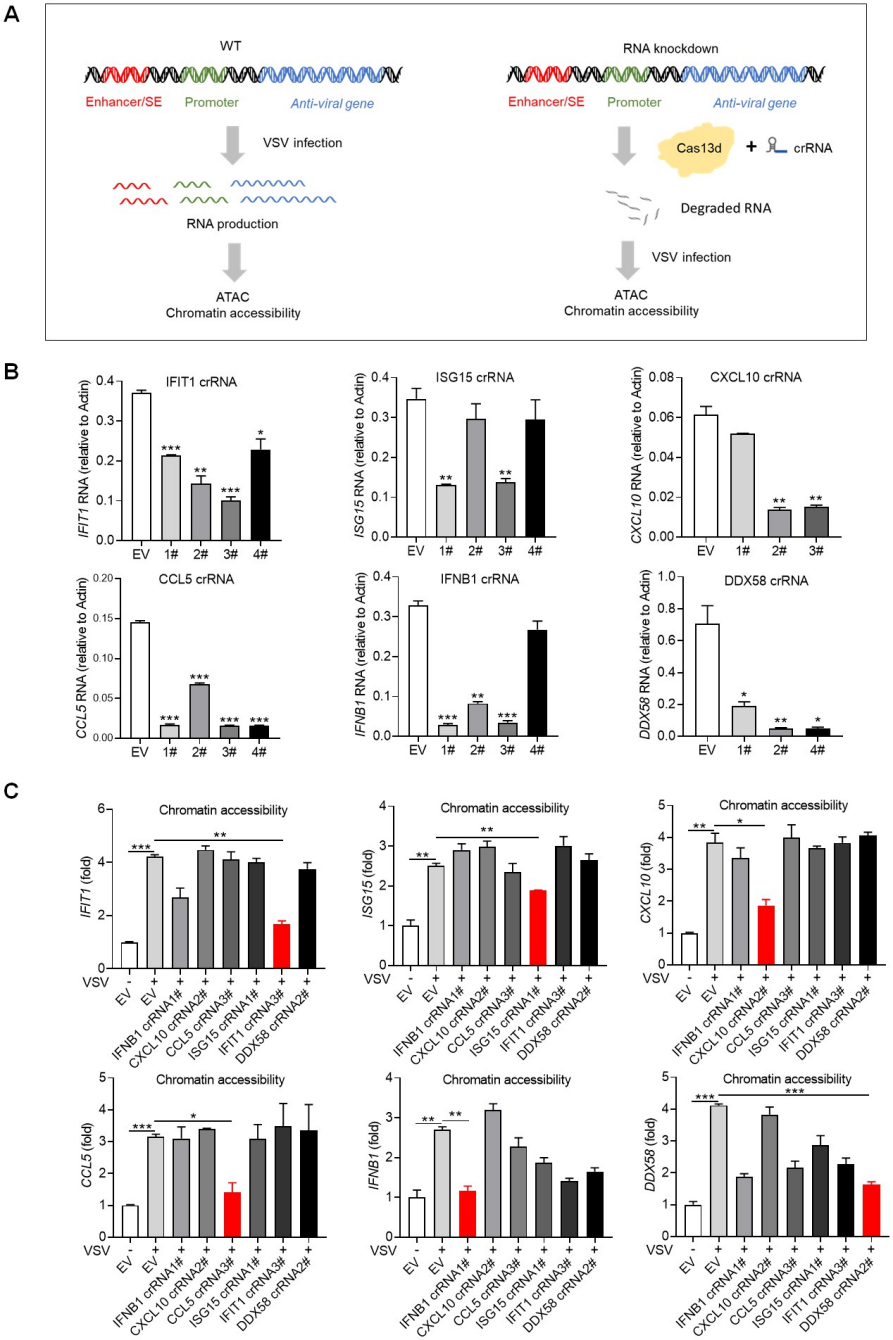
RNA product interacted with SAFA mediated specific chromatin remodeling during viral infection. (A) Models depicting the experiment design of knocking down RNA by CRISPR-Cas13d system and further detecting the chromatin accessibility with ATAC-qPCR after VSV infection. (B) Histogram showing the knockdown efficiency of crRNA of indicated RNAs after VSV infection for 18 hours. (C) ATAC-qPCR results showing the chromatin accessibility of indicated genes after the related RNA knockdown with or without VSV infection for 18 hours. Empty vector (EV) was used as control. *p < 0.05, **p < 0.01, ***p < 0.001 (Student’s t test, B and C). The cells were infected by VSV at 0.1 MOI. Data were pooled from three independent experiments (B and C). Error bars, SEM. n = 3 cultures.

To explore the potential role of SAFA-interacting RNA in regulating chromatin accessibility, we sought to knockdown the specific RNA product by CRISPR-Cas13d system and further detect the chromatin accessibility with ATAC-qPCR after viral infection (Fig 5A) [51]. Results showed that this system could induce efficient RNA knockdown after VSV infection, and we selected CRISPR RNA (crRNA) 3# for *IFIT1*, crRNA 1# for *ISG15*, crRNA 2# for *CXCL10*, crRNA 3# for *CCL5*, crRNA 1# for *IFNB1* and crRNA 2# for *DDX58* for further experiments (Fig 5B). Interestingly, cells expressing specific crRNA were not able to sustain corresponding chromatin accessible during viral infection (Fig 5C). Consistently, the corresponding mRNA expression were apparently knockdown (S5A Fig). Further, we did this experiment with crRNA targeting housekeeping genes GAPDH and α-ACTIN. ATAC-qPCR results showed that depletion of GAPDH or α-ACTIN RNA did not affect the chromatin accessibility of related genes (S5B Fig). These results suggest that RNA product interacting with SAFA after viral infection mediated the accessibility of corresponding chromatin regions.

Notably, almost all these VSV induced RNAs are known to require the RLR pathways for induction. MAVS, a key adaptor protein of RLR signaling, mediates the recruitment of downstream transcription factors NFκB and IRFs and the transcriptional activation of interferons and proinflammatory cytokine genes. We thus infected wild-type and *MAVS*^−/−^, *IRF3*^−/−^ THP-1 cells with VSV and did ATAC-qPCR. Compared with those in wild-type cells, the inducible accessibilities of *IFIT1*, *CXCL10*, *CCL5*, *IFNB1*, *DDX58* and *ISG15* were largely decreased in *MAVS*^−/−^ and *IRF3*^−/−^ cells (S5C Fig). The strong dependence of chromatin accessibility on MAVS and IRF3 supported that extranuclear signaling pathways conferred a requirement for remodeling of chromatin after viral infection.

### Virus-mediated cleavage separates the RNA-binding domain from SAFA

**Fig 6:**
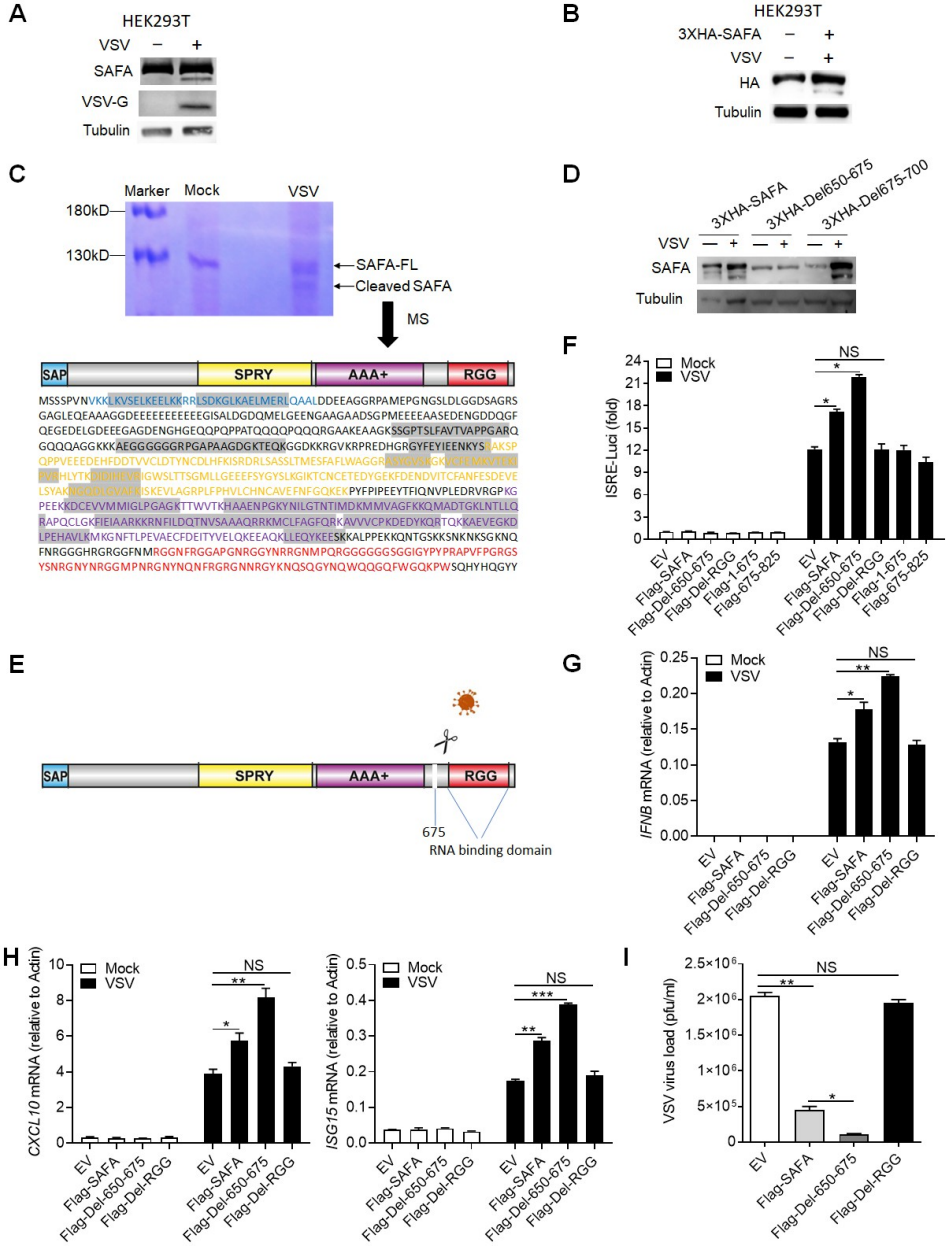
Virus-mediated cleavage of SAFA separates the RNA-binding domain. (A) Immunoblotting results showing the expression of indicated protein in HEK293T cells infected with VSV for 4 hours. (B) HEK293T cells were transfected with 3XHA-SAFA plasmids, and then infected with VSV for 4 hours followed by immunoblotting. (C) THP-1 cells were infected with VSV for 4 hours followed by immunoprecipitation and coomassie brilliant blue staining. The cleaved band was cut out for mass spectrum assay. The detected amino acid sequences were marked by grey background. (D) HEK293T cells were transfected with indicated plasmids, and then infected with VSV for 4 hours followed by immunoblotting. (E) Models depicting VSV infection induced cleavage of SAFA. (F) HEK293T cells were transfected with indicated plasmids before infection with VSV for 24 hours and then type I interferons in the supernatants were detected by bioassay. (G-I) THP-1 mutants generated by overexpressing indicated lentivirus plasmids were infected with VSV for 24 hours and the expression levels of *IFNB* (G) and *CXCL10*, *ISG15* (H) were detected by qPCR. The viral load was detected by plaque assay (I). *P < 0.05, **P < 0.01 and ***P < 0.001 (Student’s t-test). The cells were infected by VSV at 0.1 MOI. Data were representative of three independent experiments (A-D). Data were pooled from 3 independent experiments (F-I). Error bars, SEM. n = 3 cultures.

Interestingly, in VSV infected cells, we repeatedly observed a protein band just under the SAFA protein, with a smaller molecular weight of 10–20 kDa (Fig 6A and S6A Fig). Thus, we reasoned that VSV infection might promote cleavage of SAFA protein. To prove this hypothesis, we infected HEK293T cells that overexpressed N-terminal 3XHA-tagged SAFA with VSV, and a clear band similar to endogenous result was observed (Fig 6B). This result indicated that VSV infection led to a cut of SAFA at the C terminus, thus releasing the big N-terminal fragment. Further, we immunoprecipitated the cleaved band with SAFA antibody and visualized it with coomassie brilliant blue R250 staining. Then this band was cut out and sent for mass spectrometry analysis. The detected amino acid sequences located in the N-terminal SAP domain and the middle SPRY and AAA+ domain, but not the C-terminal RGG domain (Fig 6C), and the last amino acid detected was Lys^675^. Further, we constructed two 3XHA-tagged deletion mutants that deleted the 650 to 675 amino acids (3XHA-Del650-675) or the 675–700 amino acids (3XHA-Del675-700). 3XHA-Del650-675 mutant showed resistance to VSV-infection induced cleavage of SAFA, indicating that amino acids 650 to 675 were VSV targeted sequences (Fig 6D). Caspases are a group of cysteine dependent aspartate-specific proteases that has been widely implicated in many biological processes. We detected SAFA cleavage with or without pan-caspase inhibitor Z-VAD-FMK treatment and the cleaved band was obviously blocked, indicating that the host caspases were the protease responsible for cleaving SAFA (S6B Fig). Together, these results suggested that VSV infection mediated cleavage of SAFA which separated the RNA-binding domain (Fig 6E).

In agreement, HEK293T cells expressing the mutated SAFA (Flag-Del-650-675) produced more type I-IFNs compared with wild-type SAFA upon VSV infection (Fig 6F and S6C and S6D Fig). Besides, RGG domain deletion (Flag-Del-RGG) deprived SAFA of facilitating interferon production and neither the fragment 1–675 nor the fragment 675–825 could augment IFN-β activation (Fig 6F and S6C Fig), indicating that the antiviral function of SAFA was depend on the integrity of the big N-terminal fragment and the C-terminal RGG domain. Further, we expressed these mutants into THP-1 cells (S6E Fig). Compared with that in the Flag-SAFA expressing cells, the synergistic effect of SAFA-induced interferon and ISGs production after VSV infection was increased in the Flag-Del-650-675 expressing cells and decreased in the Flag-Del-RGG expressing cells (Fig 6G and 6H). Consistently, the mutated SAFA (Flag-Del-650-675) suppressed the replication of VSV more effectively (Fig 6I). Moreover, RIP-qPCR results showed that the interaction with anti-viral RNAs was significantly decreased after RGG mutation and increased after Del-650-675 mutation (S6F Fig). Consistently, ATAC-qPCR and ChIP-qPCR results also showed similar results (S6G and S6H Fig). These data collectively demonstrated the importance of RNA binding ability of SAFA in antiviral immune response.

## Discussion

Chromatin remodeling plays a central role in regulation of gene expression. The innate immune system responses rapidly to invading pathogens, which immediately produces various cytokines to eliminate the infection. The accurate and effective cytokines production can eliminate the infection without causing host pathology that is pivotal for the host. We previously reported that the nuclear matrix protein SAFA surveilled viral RNA and regulated antiviral gene expression [35]. However, how SAFA activates and regulates the expression and what determines the specificity of the induction of antiviral immune genes remains unknown. In the present study, we identified that SAFA regulated chromatin accessibility of antiviral gene and the SAFA-interacting RNA mapped the specific genomic sites. First, accumulating evidence has shown that SAFA is involved in regulation of chromatin structure from a compacted state to an active open state, indicating an important potential of SAFA in chromatin remodeling [33, 34]. Second, our genome wide sequencing results from ATAC-seq, RNA-seq and ChIP-seq in wild-type and SAFA deficient cells after VSV infection showed that SAFA was essential for chromatin accessibility, gene expression and enhancers/super-enhancers activation of antiviral genes (Figs 1 and 2). Third, in the interphase, SAFA remodels chromatin structure through oligomerization with chromatin-associated RNAs [34]. Our results suggest that the RNA binding ability of SAFA is also indispensable for its function in regulating chromatin accessibility of antiviral genes after viral infection (Fig 3). Intriguingly, VSV infection induced cleavage of SAFA that removed the RGG domain (Fig 6), indicating the importance of RNA-binding ability of SAFA in antiviral response.

Modulation of chromatin accessibility determines which gene is to be transcribed and therefore, chromatin modulation determination during viral infection is critical for host defense [14, 52]. Our results suggested that SAFA mediated the modulation of anti-viral chromatin accessibility and this process was dependent on its RNA-binding ability (Fig 3). Further RIP-seq results showed that the RNAs interacting with SAFA after viral infection were mainly antiviral related (Fig 4), and knockdown of these RNAs impaired the accessibility of specific genomic sites (Fig 5). These results indicate that the RNA products during viral infection mediated the accessibility of related genes. Rigorous regulation of cytokines production is a crucial cellular process, in which different kinds and levels of cytokines are produced at different stages of infection. RNA products are diverse and short-lived and reflect the transcriptional program directly, showing great potential in regulation of biological processes [28, 29]. There are growing evidence suggesting that RNAs play important roles in regulation of chromatin accessibility at defined genomic loci [22–24, 53]. Moreover, it has been reported that RNA products provided feedback on transcription via regulation of electrostatic interactions in transcriptional condensates [50]. SAFA protein showed multivalent interactions potential that it undergoes oligomerization after binding to RNAs during viral infection, indicating that condensates formation may occur during the activation of SAFA [54].

Combined with our previous results (35), SAFA was able to interact with both viral RNA and host RNA after virus infection. Due to the robustly increased abundance of viral RNA and double-stranded RNA structure, SAFA exhibited higher affinity to viral RNA than host RNA. It has been reported that newly synthesized RNA interacted with SAFA and promoted the oligomerization of SAFA (34). Interestingly, we here found that SAFA was essential for activation of virus inducible genes but not housekeeping genes. It is unknown how SAFA selectively facilitates the opening of antiviral genes. We here demonstrated that the host anti-viral RNA mediated the selective action of enhancers. In short, the viral RNA triggers the oligomerization of SAFA and initial transcription of a group of chromosomes associated RNA (caRNA), and host caRNA mediated selective activation of antiviral enhancers and might further amplified or maintained these responses.

To escape the inhibitory effects of host immune system, viruses have evolved various mechanisms to dampen the immune response. During DNA virus infection, inflammatory caspases cleave cGAS at the N- terminal that renders its activity in facilitating type I interferons production [55]. Cleavage of hnRNP-M is a general strategy utilized by picornaviruses to facilitate viral replication [56]. Here we report that VSV cleaved both human and mice SAFA protein, resulting in RGG domain depletion from SAFA (Fig 6).

Extranuclear signaling pathways were generally critical for anti-viral signal transduction. These signals eventually converge in the nucleus. SAFA, which predominantly localized in the nucleus, oligomerized with the generated RNA product and initiated and maintained the openness of corresponding genes. ATAC-qPCR results showed that the accessibility of antiviral immune genes was determined by the RNA product interacting with SAFA in an extranuclear signaling pathway dependent way (S5C Fig). Thus, intranuclear and extranuclear signaling pathways cooperate and form a transcriptionally responsive mesh that remodels chromosome structures and facilitates anti-viral innate immunity.

Taken together, our results provide insights into how SAFA and RNAs collaborate to reprogram specific chromatin modulation and regulate the expression of antiviral genes.

## Materials and methods

### Cells

THP-1 cell was a gift from Zhengfan Jiang (Peking University). 2fTGH-ISRE cell (human fibrosarcoma cell expressing an ISRE driven luciferase reporter) was generated by stabilizing ISRE-luciferase plasmid in 2fTGH cell. Isolation of BMDM (bone-marrow derived macrophages) was performed as described [35]. *SAFA*^–/–^, *MAVS*^–/–^, *IRF3*^–/–^THP-1 cells were constructed by CRISPR-Cas9 system as previously reported [35]. Cells were cultured in Dulbecco’s Modified Eagle’s Medium (DMEM) supplemented with 10% FBS, 100 U/mL Penicillin-Streptomycin. Cells were negative for mycoplasma.

### Viruses, antibodies, and reagents

VSV (Indiana strain) was gift from J. Rose (Yale University). Cells were infected with indicated viruses at a multiplicity of infection of 0.1 to 1 PFU. The following antibodies were used: Mouse Anti-hnRNP U (clone 3G6) antibody (sc-32315, Santa Cruz Biotechnology), Rabbit Anti-HA antibody (H3663, Sigma-Aldrich), Rabbit Anti-Histone H3 antibody—ChIP Grade (acetyl K27) (Ab4729, Abcam), Rabbit Anti-Tubulin antibody (AF7011, Affinity), Mouse anti-Histone H3 antibody (MABI0301, MBL), Rabbit Anti-DDDDK-tag antibody (anti-Flag) (PM-020, MBL), Mouse Anti-Lamin B1 (clone 3C10G12) antibody (66095-1-Ig, Proteintech). Protease inhibitor cocktail (B14001, TargetMol). Pierce Protein A/G Agarose (20422, Thermo). Lipofectamine 3000 Reagent (3000015, ThermoFisher).

### Constructs

Expression constructs generated for this study were prepared by standard molecular biology techniques and coding sequences were entirely verified. All the deletions and mutants were constructed by standard molecular biology technique. Each construct was confirmed by sequencing.

The following constructs were purchased from Addgene: pCMV-VSV-G (8454, Addgene), psPAX2 (12260, Addgene), pXR003: CasRx gRNA cloning backbone (109053, Addgene), pXR001: EF1a-CasRx-2A-EGFP (109049, Addgene).

### Type I IFN bioassay

Type I IFN bioassay was performed as previously reported [35]. Type I IFNs in human cell culture medium were quantified using a 2fTGH-ISRE cell line stably expressing an ISRE-Luci reporter. In brief, 200 mL of culture medium was incubated with confluent 2fGTH-ISRE-Luci cells (24-well plate) for 6 hours. Cells were lysed in passive lysis buffer and subjected to luciferase quantification (Promega). A serial dilution of human IFNb was included as standards.

### Luciferase reporter assay

Luciferase reporter assay was performed as previously reported [57]. HEK293T cells seeded on 24-well plates were transiently transfected with 50 ng of the luciferase reporter plasmid together with a total of equal amount of indicated expression plasmids or empty control (EV) plasmid. As an internal control, 10 ng pRL-TK was transfected simultaneously. Reporter gene activity was analyzed using the Dual-Luciferase Reporter 1000 Assay System (Promega) and measured with a TD-20/20 Luminometer (Turner Designs) according to the manufacturers’ instructions.

### Plaque assays

Viral titers from the cell culture medium were determined by plaque-forming assays as previously described (35). Briefly, virus-containing medium was serially diluted and then added to confluent Vero cells. After incubation for 1 hour, supernatants were removed, cells were washed with PBS, and culture medium containing 2% (wt/vol) methylcellulose was overlaid for 24 hours. Then cells were fixed for 30 minutes with 0.5% (vol/vol) glutaraldehyde and then stained with 1% (wt/vol) crystal violet dissolved in 70% ethanol for 30 minutes. After washing twice with ddH_2_O, plaques were counted, and average counts were multiplied by the dilution factor to determine the viral titer as plaque-forming units per milliliter.

### Western blotting

Cells were harvested and lysed with Pierce lysis buffer (25 mM Tris·HCl, pH 7.4, 150 mM NaCl, 1 mM EDTA, 1% NP-40, 5% β-Mercaptoethanol) with the protease inhibitor cocktail (Roche) on ice for 30 minutes. Supernatants were collected by centrifugation at 12,000 rpm for 10 minutes at 4°C. Cell lysates were boiled with loading buffer. Each protein sample was loaded onto 8% SDS-PAGE. After electrophoresis, proteins were transferred to the nitrocellulose membrane (Millipore). The membrane was blocked with 5% milk (in PBST) for 1 hour, and incubated sequentially with primary and HRP-coupled secondary antibodies. After being washed with PBST for 3 times, the membranes were visualized by enhanced chemiluminescence (Millipore).

### Coomassie brilliant blue staining

Samples pulled down with SAFA antibody were anaylzed with SDS-PAGE. After staining with Coomassie brilliant blue R-250, the target bands on the PAGE gel were visualized and excised for mass spectrometry.

### RNA knockdown

RNA knockdown was performed by CRISPR-Cas13d system. Specific CRISPR RNAs (crRNAs) were annealed and ligated into CasRx gRNA cloning backnone (addgene, #109053). CrRNA plasmids (2 ug) and plasmids coding CasRx (addgene, #109049) were transfected into HEK293T cells together (6-well plate). The medium was changed to fresh DMEM containing 10% FBS at 6 hours post transfection. After transfection for 48 hours, GFP-highly positive cells were sorted by using fluorescence-activated cell sorting (FACS) and used for further experiment. The crRNAs used were listed in the S1 Table.

### Quantitative real-time PCR (qRT-PCR)

Total RNA was isolated using the RNA simple Total RNA kit (TIANGEN). 1 ug RNA was reverse transcribed using a FastKing RT Kit (TIANGEN). Levels of the indicated genes were analyzed by qRT-PCR amplified using SYBR Green (Transgene). Data shown are the relative abundance of the indicated mRNA normalized to *Actin*. The primers used were listed in the S2 Table.

### ATAC-seq and ATAC-qPCR

Pellet 50,000 viable sample cells at 500 RCF at 4°C for 5 min. Aspirate all supernatant. Add 50 μL cold ATAC-Resuspension Buffer (RSB) containing 0.1% NP40, 0.1% Tween-20, and 0.01% Digitonin into the cell pellet and pipette up and down 3 times. Incubate on ice for 3 minutes. Wash out lysis with 1 mL cold ATAC-RSB containing 0.1% Tween-20 but no NP40 or digitonin and invert tube 3 times to mix. Pellet nuclei at 500 RCF for 10 min at 4°C. Aspirate all supernatant. Resuspend cell pellet in 50 μL of transposition mixture (25 μL 2x TD buffer, 2.5 μL transposase (100 nM final), 16.5 μL PBS, 0.5 μL 1% digitonin, 0.5 μL 10% Tween-20, 5 μL H_2_O) by pipetting up and down 6 times. Incubate reaction at 37°C for 30 minutes. Afterward, the DNA was purified with Magen DNA purify kit and amplified with primers containing barcodes by using the TruePrep DNA Library Prep Kit (TD501-01). Subsequent sequencing and data analysis were handed over to GENEWIZ Biotechnology Co., LTD (Suzhou, China). Briefly, all libraries were adapted for high-throughput sequencing (75 bp paired-end) on an Illumina NextSeq 500. Raw sequencing data are collected. After filtering data through sequencing data quality assessment, clean reads are further obtained. After removing adapter sequences and low-quality reads, high-quality reads are processed for further analysis. The peak calling reads are mapped to the human genome and accessible chromatin regions, such as promoters, 3’ UTR and 5’ UTR. The data were first merged by the “bedtools merge” tool (https://bedtools.readthedocs.io/en/latest/content/tools/merge.html). Then all the data were normalized by the “bamCoverage” tool with the RPKM parameter (https://deeptools.readthedocs.io/en/develop/content/tools/bamCoverage.html). The normalized RPKM were used for further analysis. The original data of the ATAC-seq was uploaded to the GEO DataSets (https://www.ncbi.nlm.nih.gov/geo/query/acc.cgi?acc=GSE199672; https://www.ncbi.nlm.nih.gov/geo/query/acc.cgi?acc=GSE199827).

For ATAC-qPCR, the library construction is the same as the method in ATAC-seq. Then the ATAC libraries were adapted for qRT-PCR with specific primers. Primers are designed according to previous articles [58]. The primers used in this article were listed in the S3 Table.

### RNA-seq

Whole RNA of cells with specific treatment were purified using RNeasy Mini Kit (QIAGEN NO. 74104). The transcriptome library for sequencing was generated using VAHTSTM mRNA-seq v2 Library Prep Kit for Illumina (Vazyme Biotech, Nanjing, China) following the manufacturer’s recommendations. After clustering, the libraries were sequenced on Illumina Hiseq X Ten platform using (2 3 150bp) paired-end module. The raw images were transformed into raw reads by base calling using CASAVA (https://support.illumina.com.cn/sequencing/sequencing_software/casava.html). Then, raw reads in a fastq format were first processed using inhouse perl scripts. Clean reads were obtained by removing reads with adapters, reads in which unknown bases were more than 5% and low-quality reads (the percentage of low quality bases was over 50% in a read, we defined the low quality base to be the base whose sequencing quality was no more than 10). At the same time, Q20, Q30, GC content of the clean data were calculated (Vazyme Biotech, Nanjing, China). For analysis, the data was normalized by the FPKM to eliminate the gene length and sequencing depth effects. Then we compared the differences between wild-type and mutated cells with or without viral infection for indicated times. The original data of the RNA-seq was uploaded to the GEO DataSets (https://www.ncbi.nlm.nih.gov/geo/query/acc.cgi?acc=GSE199674).

### RIP-seq

For RNA immunoprecipitation sequencing (RIP-Seq), six 10-cm^2^ dishes (1x10^7^ cells/dish) of HEK293 cells were infected with VSV. Then washed with ice cold PBS, scrapped and pelleted at 2500 rpm for 5 min at 4°C. After lysed in Pierce lysis buffer containing protease and RNase inhibitors for 30 min at 4°C, supernatants were collected by centrifugation at 12,000 rpm for 10 min at 4°C. 6ug anti-SAFA antibody were added to the supernatant and incubated for 2 h at 4°C with gentle rotation. Further, protein A/G beads were added and incubated for 1 h at 4°C with gentle rotation. After incubation, beads were pelleted at 2,500 rpm for 30 s and washed for a total of four times: three times with lysis buffer and the last time with pre-cold PBS. Then the co-precipitated RNAs were isolated according to manufacturer’s instructions. The transcriptome library for sequencing was generated and sequenced on Illumina Hiseq X Ten platform using (2x150bp) paired-end module. The sequenced data were first normalized by RPKM. Then the data of all the experimental groups were compared to the data of their relative input group using the “bamCompare” tool (https://deeptools.readthedocs.io/en/develop/content/tools/bamCompare.html). The compared data were used for further analysis. We compared the differences between wild-type and mutated cells with or without viral infection for indicated times. The original data of the RIP-seq was uploaded to the GEO DataSets (https://www.ncbi.nlm.nih.gov/geo/query/acc.cgi?acc=GSE199677).

### ChIP-seq and ChIP-qPCR

Chromatin immunoprecipitation followed by sequencing (ChIP-seq) and data analysis were conducted as previously described [35]. Approximately 10^7^ treated cells were crosslinked with 1% formaldehyde at room temperature for 10 min, and the reaction was quenched with 0.125M glycine for 5 min. The cells were washed twice with PBS, then scrapped and pelleted at 2500 rpm for 5 min at 4°C. After lysis and sonication, the purified DNA sizes were analyzed by 2% agarose gel electrophoresis. The majority of the sonicated DNA fragments were sheared to a size of around 200–600 bp. The sonicated chromatin was spun down at 12,000 rpm for 10 min at 4°C to collect the chromatin. Then the soluble chromatin was incubated with 2–5 mg of antibodies and the mixture were rotated at 4°C overnight. After incubation, pre-washed Protein G Dynabeads (10004d, Invitrogen) were added and incubated for 4 h at 4°C in a rotator. Then the magnetic Dynabeads were pelleted by placing the tubes in a magnetic rack and were washed for a total of five times: once with wash buffer A (20 mM Tris-HCl (pH 8.0), 500 mM NaCl, 2 mM EDTA, 0.1% SDS, 1% Triton X-100); once with wash buffer B (10 mM Tris-HCl (pH 8.0), 250 mM LiCl, 1 mM EDTA, 1%NP-40); three times with wash buffer C (1 mM EDTA, 10 mM Tris-HCl (pH 8.0)). After the last wash, beads were resuspended in 100 mL elution buffer (50 mM Tris-HCl (pH 8.0), 10 mM EDTA, 1% SDS), followed by incubation at 65°C overnight for reverse crosslink. The next day, purify DNA with QIA quick PCR purification kit (Magen, D211102) and elute with 50 mL elution buffer. The extracted DNA were send for sequencing. The sequenced data were first normalized by RPKM. Then the data of all the experimental groups were compared to the data of their relative input group using the “bamCompare” tool (https://deeptools.readthedocs.io/en/develop/content/tools/bamCompare.html). The compared data were used for further analysis. We compared the differences between wild-type and mutated cells with or without viral infection for indicated times. The original data of the ChIP-seq was uploaded to the GEO DataSets (https://www.ncbi.nlm.nih.gov/geo/query/acc.cgi?acc=GSE199402).

For ChIP-qPCR, the library construction is the same as the method in ChIP-seq. Then the libraries were adapted for qRT-PCR with specific primers. The primers used in this article were listed in the S4 Table.

### Statistical analysis

For all the bar graphs, data were expressed as means ± SEM. Prism 8 software (graphic software) was used for charts, and statistical analyses. Differences in means were considered statistically significant at p < 0.05. Significance levels are: * *p* < 0.05; ** *p* < 0.01; *** *p* < 0.001; **** *p* < 0.0001; NS., non-significant.

## Supporting information

S1 Fig SAFA deficiency decreased the chromatin accessibility of antiviral immune genes. (A) Models depicting the ATAC-seq and RNA-seq in Wild-type (WT) and *SAFA*^−/−^ THP-1 cells with VSV infection(upper), and immunoblotting results showing the knockout of SAFA in THP-1 cells (lower). (B) Feature distribution of ATAC-seq profile after VSV infection in WT and *SAFA*^−/−^ THP-1 cells. (C) Line graph showing SAFA in regulation of VSV induced accessible locus and insensitive locus. (D) Violin graph showing ISGs affected by SAFA depletion in ATAC-seq. (E) Genome browser views of ATAC-seq signal for the indicated genes. (F) WT and *SAFA*^−/−^ THP-1 cells were infected with VSV infection for indicated times, and ATAC-qPCR showed the chromatin accessibility of indicated genes. (G) GO term enrichment analysis of genes significantly affected by SAFA depletion in RNA-seq. (H) Counting Kit-8 (CCK-8) assay to evaluate the cell viability at indicated time points infected by VSV at 0.1 MOI in both HEK293T cells and THP-1 cells. *p < 0.05, **p < 0.01, ***p < 0.001 ****p < 0.0001 (Student’s t test; D, F and H). Data were pooled from two independent experiments (B, C and E).
(TIF)

S2 Fig SAFA deficiency decreased the activation of antiviral immune genes. (A) Heatmap showing the ChIP-seq signal enrichment around the TSSs of H3K27ac in WT and *SAFA*^−/−^ THP-1 cells with VSV infection for 8 or 24 hours. (B) Histogram diagram showing amounts of enhancers in WT and *SAFA*^−/−^ THP-1 cells with VSV infection. (C) Histogram diagram showing amounts of super-enhancers in WT and *SAFA*^−/−^ THP-1 cells with VSV infection. (D) Genome browser views of ChIP -seq signal for the indicated genes. (E) WT and *SAFA*^−/−^ THP-1 cells were infected with VSV infection for indicated times, and ChIP-qPCR signal showing H3K27Ac occupancy of indicated genes. (F) WT and *SAFA*^−/−^ THP-1 cells were infected with VSV infection for indicated times, and ChIP-qPCR signal showing RNA Ploymerase II occupancy of indicated genes. (G) Pie graph showing distribution of super-enhancer-driven genes. *p < 0.05, **p < 0.01, ***p < 0.001, (Student’s t test; E and F). Data were pooled from two independent experiments (A-D).
(TIF)

S3 Fig RNA binding activity of SAFA is critical for increasing the accessibility of anti-viral chromatin. (A) Immunoblotting results showing the expression of SAFA in WT, *SAFA*^−/−^ and Flag-SAFA or Flag-Del-RGG stable-expressed *SAFA*^−/−^ THP-1 cells (left); Flag-SAFA or Flag-Del-RGG stable-expressed *SAFA*^−/−^ THP-1 cells were infected with VSV, with or without RNAase treatment, and then resolved by Native Page (right). (B) Feature distribution of ATAC-seq profile after VSV infection. (C) Genome browser views of ATAC-seq signal for the indicated genes. (D) Flag-SAFA or Flag-Del-RGG stable-expressed *SAFA*^−/−^ THP-1 cells were infected with VSV infection for indicated times, and ATAC-qPCR showed the chromatin accessibility of indicated genes. (E) Violin graph showing ISGs affected by RGG domain depletion in ATAC-seq. (F) GO term enrichment analysis of genes significantly affected by RGG domain depletion in RNA-seq. *p < 0.05, **p < 0.01, ***p < 0.001 (Student’s t test; C). Data were pooled from two independent experiments (D and E). Data were representative of two independent experiments (A-C).
(TIF)

S4 Fig SAFA interacted with antiviral related RNAs in a time-dependent manner during viral infection. (A) Models depicting the RIP-seq assay of SAFA in THP-1 cells with VSV infection for 6 or 24 hours. (B) Line graph showing time-dependent RNA binding manner of indicated genes with VSV infection for indicated times.
(TIF)

S5 Fig SAFA-interacting RNA mediated specific chromatin remodeling in an extranuclear pathway dependent manner. (A) Histogram showing the RNA expression with indicated crRNA transfection for 48 hours and with or without VSV infection for 18 hours. (B) Histogram showing the the knockdown efficiency of crRNA of indicated RNAs after VSV infection for 18 hours (left); ATAC-qPCR results showing the chromatin accessibility of indicated genes after the related RNA knockdown with or without VSV infection for 18 hours (right). (C) ATAC-qPCR results showing the chromatin accessibility of indicated genes after VSV infection for 18 hours in WT, *IRF3*^−∕−^ and *MAVS*^−∕−^ THP-1 cells. *p < 0.05, **p < 0.01, ***p < 0.001 (Student’s t test). Data were pooled from three independent experiments. Error bars, SEM. n = 3 cultures.
(TIF)

S6 Fig Virus-mediated cleavage separates the RNA-binding domain from SAFA. (A) Bone marrow derived microphage (BMDM) cells were infected with VSV for 4 hours, and the indicated protein were detected by immunoblotting. (B) HEK293T cells were pretreated with caspase inhibitor Z-VAD-FMK for 2 hours and infected with VSV for 4 hours, and the indicated protein were detected by immunoblotting. (C) HEK293T cells were transfected with indicated plasmids, and the expression level of these plasmids were detected by immunoblotting. (D) Luciferase activity of IFNβ in HEK293T cells expressing IFNβ–Luc plasmid together with either an empty vector or indicated plasmids, after 24 hours infected with VSV for 24 hours. (E) THP-1 mutants were generated by overexpressing indicated lentivirus plasmids, and the expression level of these plasmids were detected by immunoblotting. (F-H) THP-1 mutants generated by overexpressing indicated lentivirus plasmids were infected with VSV for 18 hours, and the RNA-binding ability (F), enhancer activity showed by H3K27Ac occupancy (G) and chromatin accessibility (H) of indicated genes were detected by RIP-qPCR, ChIP-qPCR and ATAC-qPCR. *p < 0.05, **p < 0.01, ***p < 0.001 (Student’s t-test). Data were representative of three independent experiments (A-D and F-H). Data were pooled from 3 independent experiments (E). Error bars, SEM. n = 3 cultures.
(TIF)

S1 Table CrRNA sequence. (DOCX)

S2 Table Primers for qRT-PCR. (DOCX)

S3 Table Primers for ATAC-qRT-PCR. (DOCX)

S4 Table Primers for ChIP-qRT-PCR. (DOCX)
