# Supplementary figures and images for: SAFA facilitates chromatin opening of immune genes through interacting with anti-viral host RNAs
Source: PLoS Pathog. 2022 Jun 3;18(6):e1010599. doi: 10.1371/journal.ppat.1010599 (PMC9200321; doi:10.1371/journal.ppat.1010599)

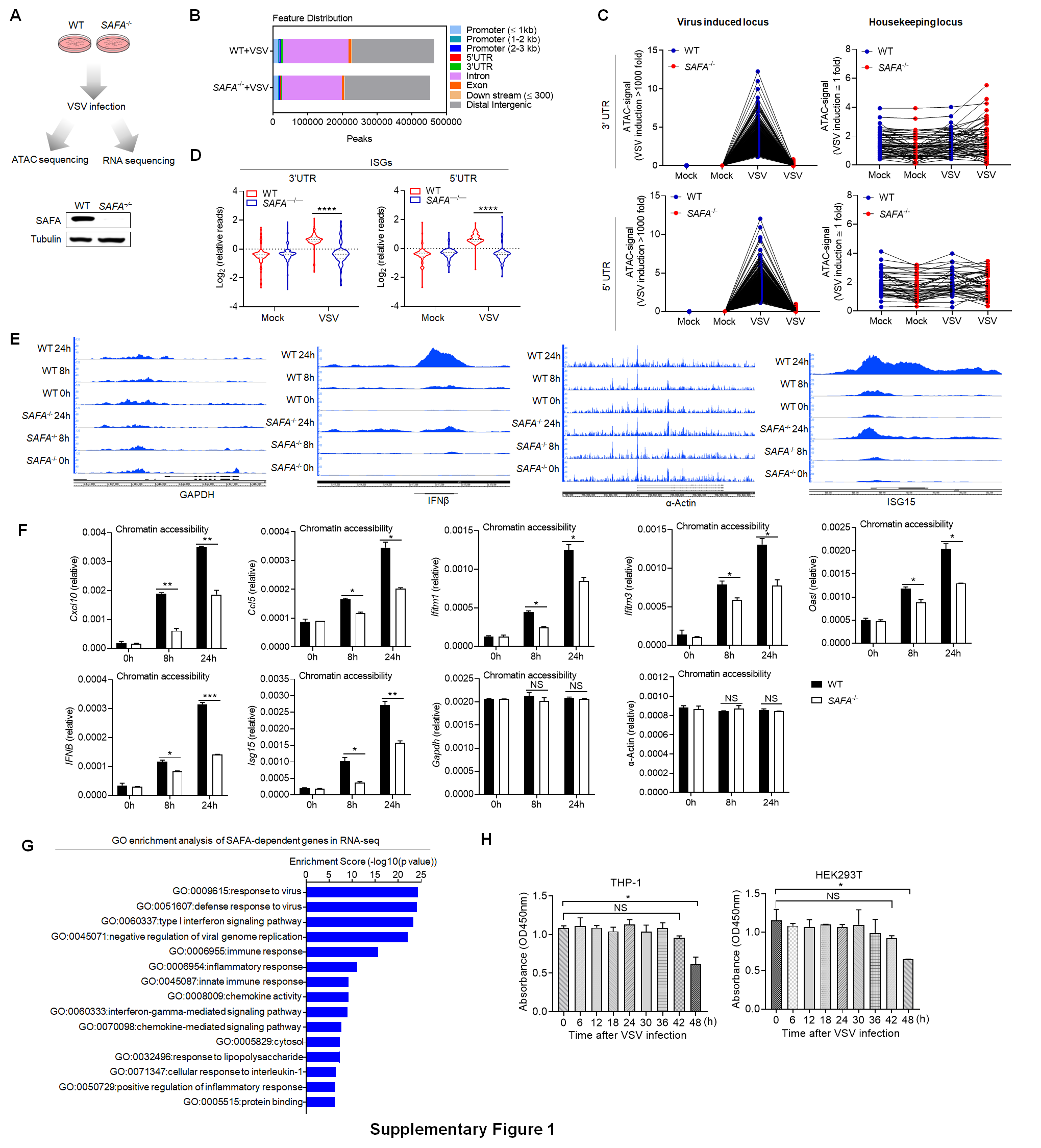

Supplement: S1 Fig — (A) Models depicting the ATAC-seq and RNA-seq in Wild-type (WT) and SAFA−/− THP-1 cells with VSV infection(upper), and immunoblotting results showing the knockout of SAFA in THP-1 cells (lower). (B) Feature distribution of ATAC-seq profile after VSV infection in WT and SAFA−/− THP-1 cells. (C) Line graph showing SAFA in regulation of VSV induced accessible locus and insensitive locus. (D) Violin graph showing ISGs affected by SAFA depletion in ATAC-seq. (E) Genome browser views of ATAC-seq signal for the indicated genes. (F) WT and SAFA−/− THP-1 cells were infected with VSV infection for indicated times, and ATAC-qPCR showed the chromatin accessibility of indicated genes. (G) GO term enrichment analysis of genes significantly affected by SAFA depletion in RNA-seq. (H) Counting Kit-8 (CCK-8) assay to evaluate the cell viability at indicated time points infected by VSV at 0.1 MOI in both HEK293T cells and THP-1 cells. *p < 0.05, **p < 0.01, ***p < 0.001 ****p < 0.0001 (Student’s t test; D, F and H). Data were pooled from two independent experiments (B, C and E). (TIF) [file ppat.1010599.s001.tif]

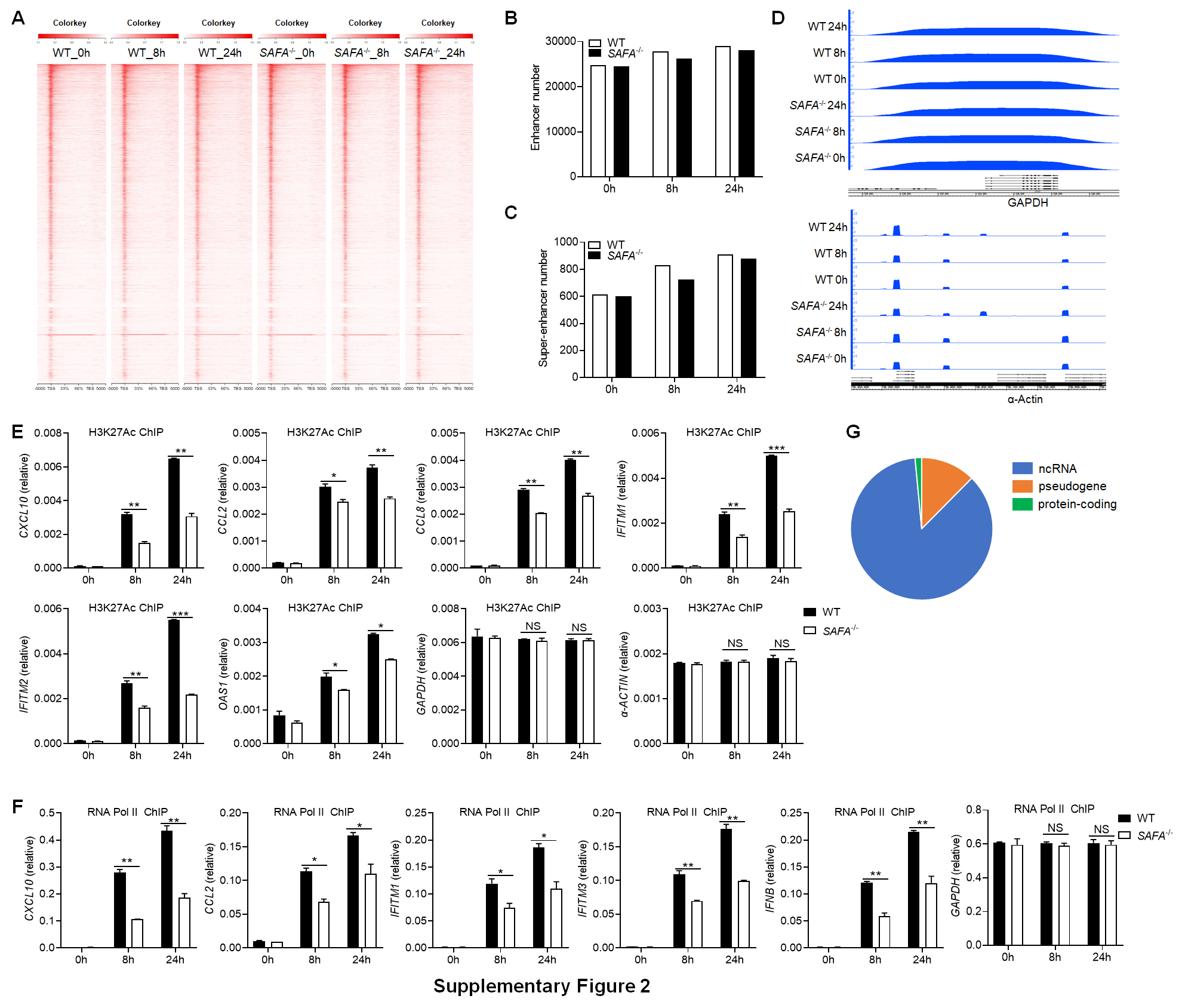

Supplement: S2 Fig — (A) Heatmap showing the ChIP-seq signal enrichment around the TSSs of H3K27ac in WT and SAFA−/− THP-1 cells with VSV infection for 8 or 24 hours. (B) Histogram diagram showing amounts of enhancers in WT and SAFA−/− THP-1 cells with VSV infection. (C) Histogram diagram showing amounts of super-enhancers in WT and SAFA−/− THP-1 cells with VSV infection. (D) Genome browser views of ChIP -seq signal for the indicated genes. (E) WT and SAFA−/− THP-1 cells were infected with VSV infection for indicated times, and ChIP-qPCR signal showing H3K27Ac occupancy of indicated genes. (F) WT and SAFA−/− THP-1 cells were infected with VSV infection for indicated times, and ChIP-qPCR signal showing RNA Ploymerase II occupancy of indicated genes. (G) Pie graph showing distribution of super-enhancer-driven genes. *p < 0.05, **p < 0.01, ***p < 0.001, (Student’s t test; E and F). Data were pooled from two independent experiments (A-D). (TIF) [file ppat.1010599.s002.tif]

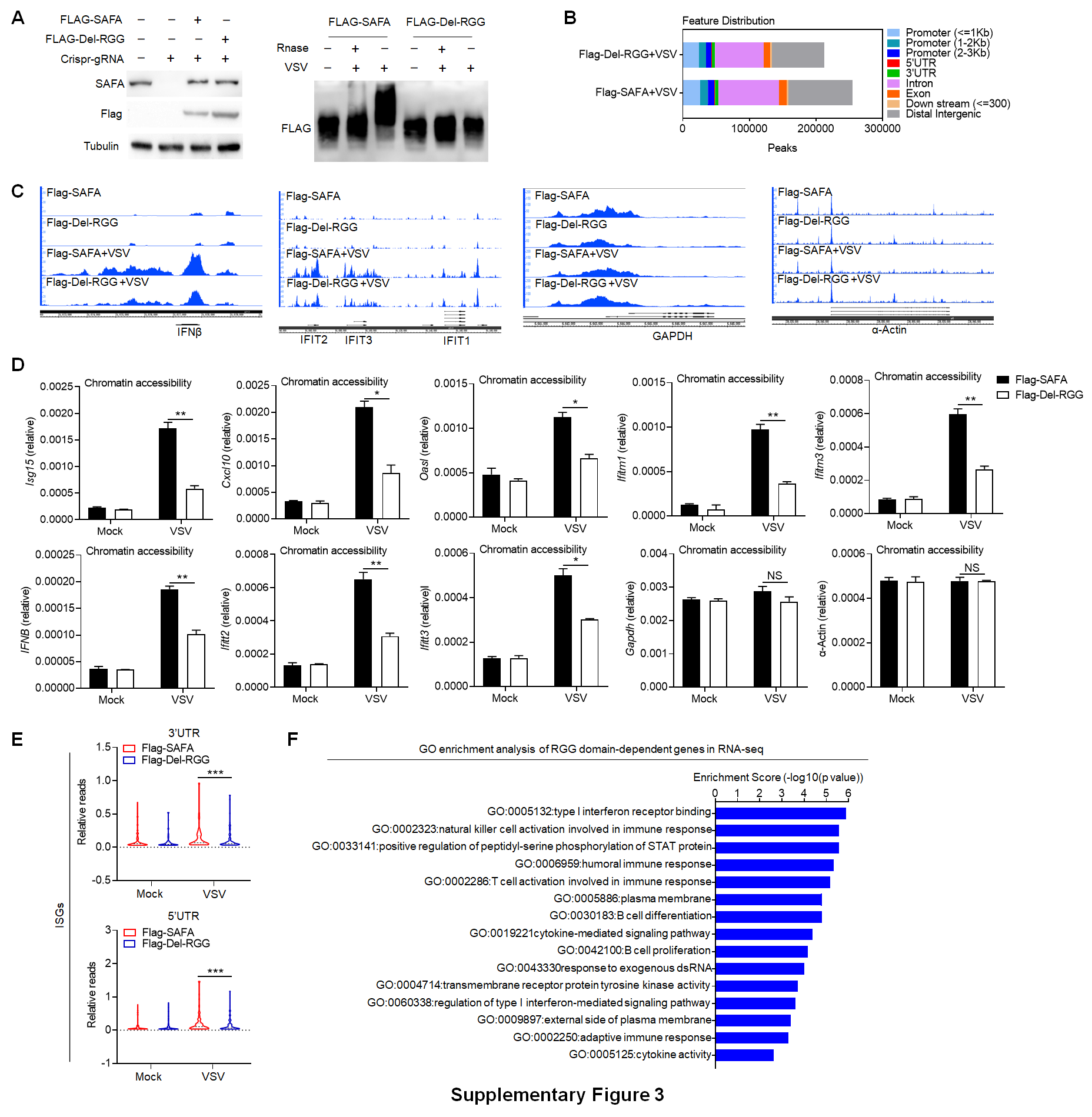

Supplement: S3 Fig — (A) Immunoblotting results showing the expression of SAFA in WT, SAFA−/− and Flag-SAFA or Flag-Del-RGG stable-expressed SAFA−/− THP-1 cells (left); Flag-SAFA or Flag-Del-RGG stable-expressed SAFA−/− THP-1 cells were infected with VSV, with or without RNAase treatment, and then resolved by Native Page (right). (B) Feature distribution of ATAC-seq profile after VSV infection. (C) Genome browser views of ATAC-seq signal for the indicated genes. (D) Flag-SAFA or Flag-Del-RGG stable-expressed SAFA−/− THP-1 cells were infected with VSV infection for indicated times, and ATAC-qPCR showed the chromatin accessibility of indicated genes. (E) Violin graph showing ISGs affected by RGG domain depletion in ATAC-seq. (F) GO term enrichment analysis of genes significantly affected by RGG domain depletion in RNA-seq. *p < 0.05, **p < 0.01, ***p < 0.001 (Student’s t test; C). Data were pooled from two independent experiments (D and E). Data were representative of two independent experiments (A-C). (TIF) [file ppat.1010599.s003.tif]

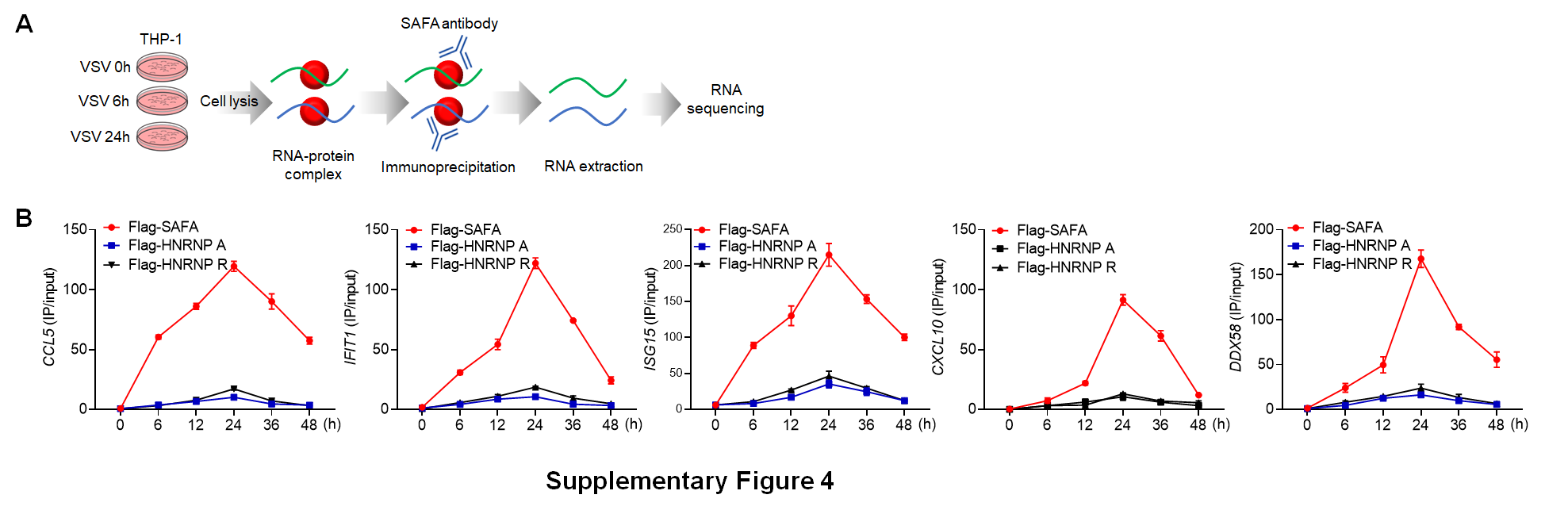

Supplement: S4 Fig — (A) Models depicting the RIP-seq assay of SAFA in THP-1 cells with VSV infection for 6 or 24 hours. (B) Line graph showing time-dependent RNA binding manner of indicated genes with VSV infection for indicated times. (TIF) [file ppat.1010599.s004.tif]

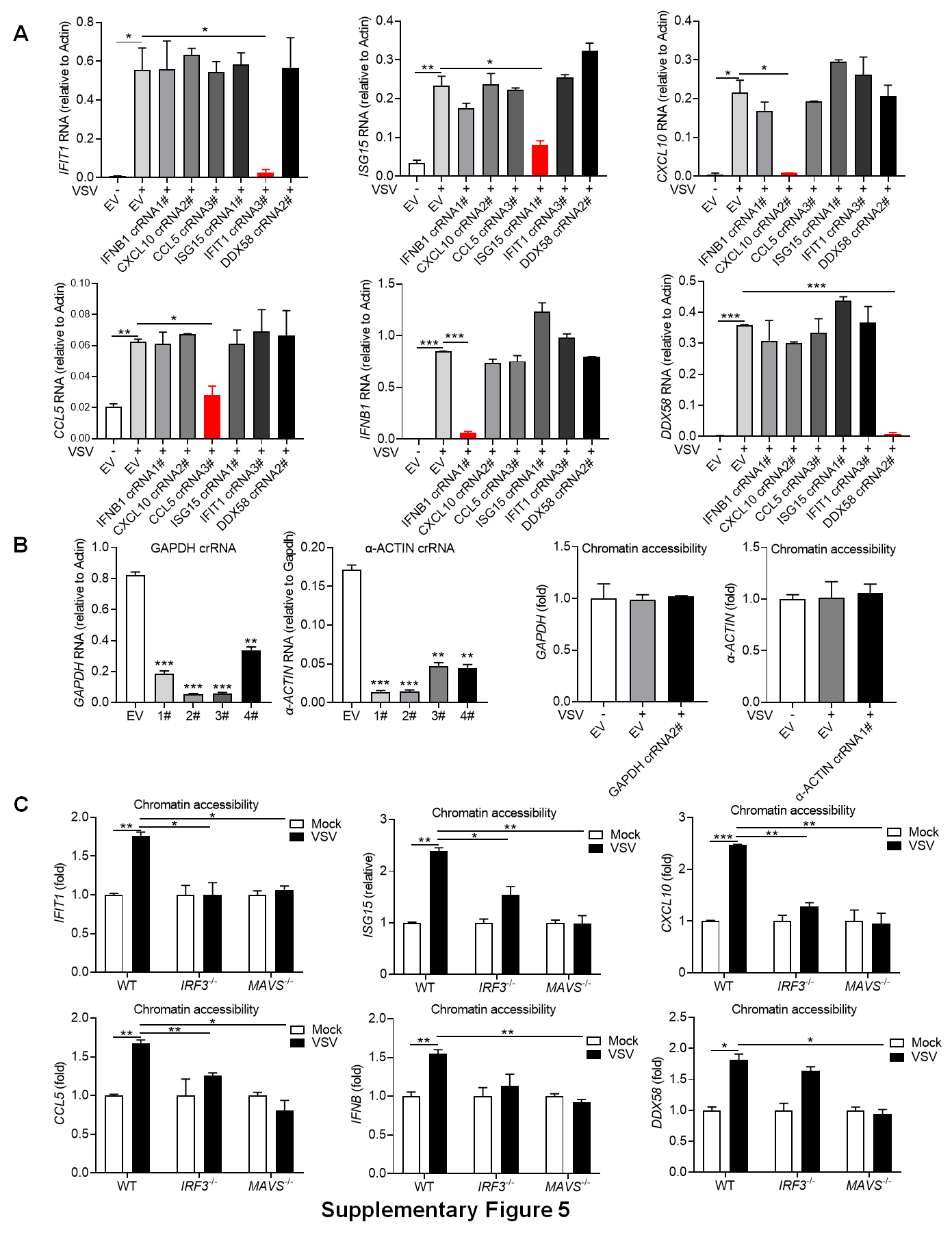

Supplement: S5 Fig — (A) Histogram showing the RNA expression with indicated crRNA transfection for 48 hours and with or without VSV infection for 18 hours. (B) Histogram showing the the knockdown efficiency of crRNA of indicated RNAs after VSV infection for 18 hours (left); ATAC-qPCR results showing the chromatin accessibility of indicated genes after the related RNA knockdown with or without VSV infection for 18 hours (right). (C) ATAC-qPCR results showing the chromatin accessibility of indicated genes after VSV infection for 18 hours in WT, IRF3−∕− and MAVS−∕− THP-1 cells. *p < 0.05, **p < 0.01, ***p < 0.001 (Student’s t test). Data were pooled from three independent experiments. Error bars, SEM. n = 3 cultures. (TIF) [file ppat.1010599.s005.tif]

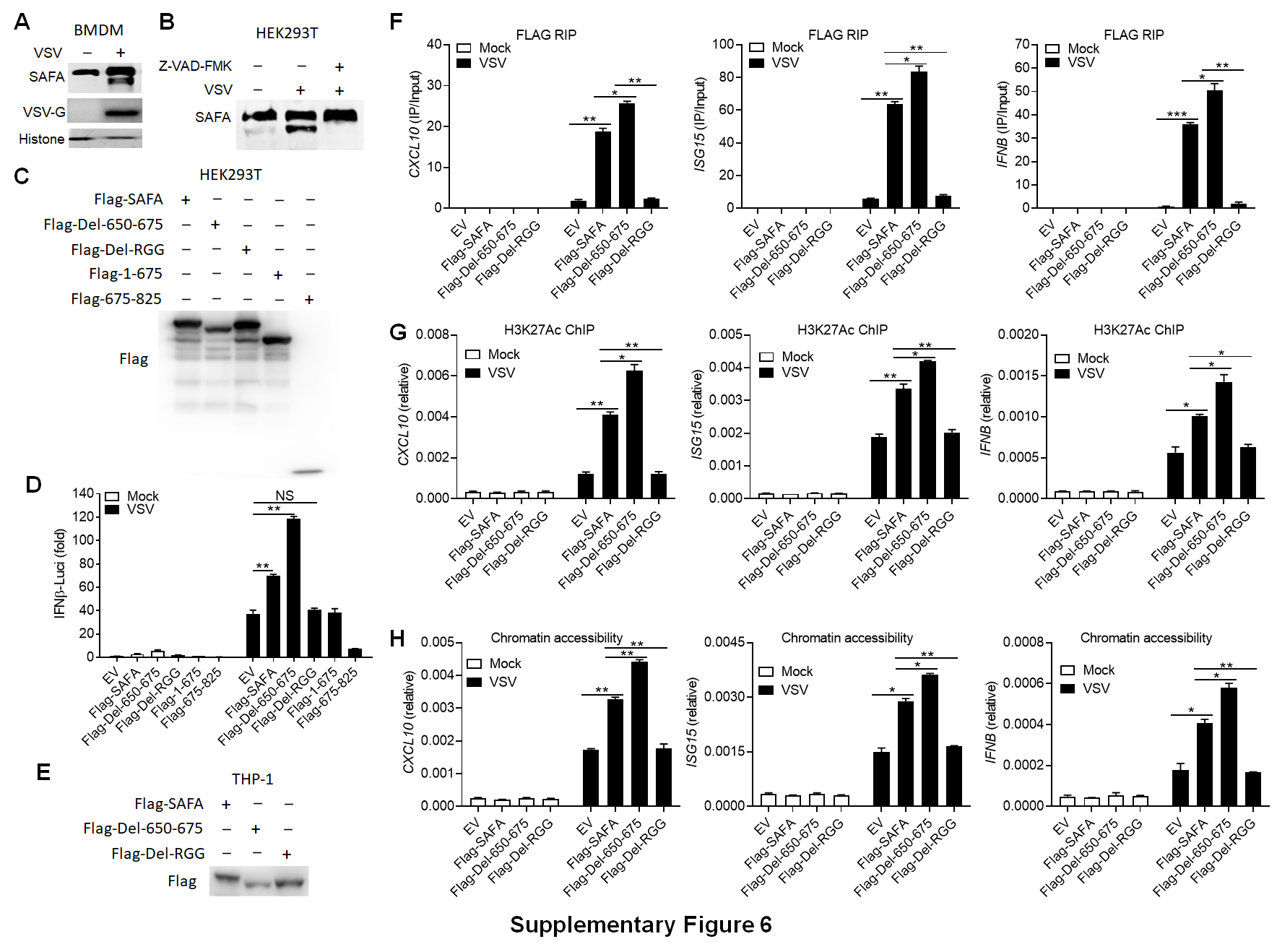

Supplement: S6 Fig — (A) Bone marrow derived microphage (BMDM) cells were infected with VSV for 4 hours, and the indicated protein were detected by immunoblotting. (B) HEK293T cells were pretreated with caspase inhibitor Z-VAD-FMK for 2 hours and infected with VSV for 4 hours, and the indicated protein were detected by immunoblotting. (C) HEK293T cells were transfected with indicated plasmids, and the expression level of these plasmids were detected by immunoblotting. (D) Luciferase activity of IFNβ in HEK293T cells expressing IFNβ–Luc plasmid together with either an empty vector or indicated plasmids, after 24 hours infected with VSV for 24 hours. (E) THP-1 mutants were generated by overexpressing indicated lentivirus plasmids, and the expression level of these plasmids were detected by immunoblotting. (F-H) THP-1 mutants generated by overexpressing indicated lentivirus plasmids were infected with VSV for 18 hours, and the RNA-binding ability (F), enhancer activity showed by H3K27Ac occupancy (G) and chromatin accessibility (H) of indicated genes were detected by RIP-qPCR, ChIP-qPCR and ATAC-qPCR. *p < 0.05, **p < 0.01, ***p < 0.001 (Student’s t-test). Data were representative of three independent experiments (A-D and F-H). Data were pooled from 3 independent experiments (E). Error bars, SEM. n = 3 cultures. (TIF) [file ppat.1010599.s006.tif]
