# Supplementary material for: SAFA facilitates chromatin opening of immune genes through interacting with anti-viral host RNAs
Source: PLoS Pathog. 2022 Jun 3;18(6):e1010599. doi: 10.1371/journal.ppat.1010599 (PMC9200321; doi:10.1371/journal.ppat.1010599)
Supplement: S1 Table — (DOCX) [file ppat.1010599.s007.docx]

S1 Table: CrRNA sequence

| Gene | crRNA |
| --- | --- |
| Human *IFB1* 1# | ATAGCAAAGATGTTCTGGAGCAT |
| Human *IFB1* 2# | AGCAAAGATGTTCTGGAGCATCT |
| Human *IFB1* 3# | AACAATAGTCTCATTCCAGCCAG |
| Human *IFB1* 4# | CTGATGATAGACATTAGCCAGGA |
| Human *CXCL10* 1# | AGTCAGAAAGATAAGGCAGCAAA |
| Human *CXCL10* 2# | GAGTCAGAAAGATAAGGCAGCAA |
| Human *CXCL10* 3# | AGAGTCAGAAAGATAAGGCAGCA |
| Human *IFIT1* 1# | AGTGACATCTCAATTGCTCCAGA |
| Human *IFIT1* 2# | GTGACATCTCAATTGCTCCAGAC |
| Human *IFIT1* 3# | AAGTGACATCTCAATTGCTCCAG |
| Human *IFIT1* 4# | GTCATCAATGGATAACTCCCATG |
| Human *ISG15* 1# | TTCGTCGCATTTGTCCACCACCA |
| Human *ISG15* 2# | TCGTCGCATTTGTCCACCACCAG |
| Human *ISG15* 3# | CGTCGCATTTGTCCACCACCAGC |
| Human *ISG15* 4# | GTTCGTCGCATTTGTCCACCACC |
| Human *DDX58* 1# | ATCCAAAAAGCCACGGAACCAGC |
| Human *DDX58* 2# | AGAAAAAGTGTGGCAGCCTCCAT |
| Human *DDX58* 4# | CATCCAAAAAGCCACGGAACCAG |
| Human *CCL5* 1# | CAAAGAGTTGATGTACTCCCGAA |
| Human *CCL5* 2# | CCAAAGAGTTGATGTACTCCCGA |
| Human *CCL5* 3# | CAAGCTAGGACAAGAGCAAGCAG |
| Human *CCL5* 4# | AAGAGCAAGCAGAAACAGGCAAA |
| Human *GAPDH* 1# | GGTTGCAACATGGCGGCCGCTCT |
| Human *GAPDH* 2# | TTCCCCGCCACACGCGACTCCAC |
| Human *GAPDH* 3# | AGCATCACCCGGAGGAGAAATCG |
| Human *GAPDH* 4# | GGCAACAATATCCACTTTACCAG |
| Human *α-ACTIN* 1# | CCAGGCTGGGTCCAGGAGCAGGT |
| Human *α-ACTIN* 2# | TCCTCGATGTTCTCAATCTGGGT |
| Human *α-ACTIN* 3# | GTTTGGGCAGCCTTTCCCCTGAG |
| Human *α-ACTIN* 4# | GAAATATCCTGAATAGCAAAGCG |
