# Supplementary material for: SAFA facilitates chromatin opening of immune genes through interacting with anti-viral host RNAs
Source: PLoS Pathog. 2022 Jun 3;18(6):e1010599. doi: 10.1371/journal.ppat.1010599 (PMC9200321; doi:10.1371/journal.ppat.1010599)
Supplement: S2 Table — (DOCX) [file ppat.1010599.s008.docx]

S2 Table. Primers for qRT-PCR

| Gene | Forward Primer (5’-3’) | Reverse Primer (5’-3’) |
| --- | --- | --- |
| Human *IFB1* | AGGACAGGATGAACTTTGAC | TGATAGACATTAGCCAGGAG |
| Human *IFIT1* | CATCAGGTCAAGGATAGTCTGGAGC | GGTTGTCATGTTCTTCCTGCATT |
| Human *ISG15* | ATCGGCGTGCACGCCTTCCAGCA | TGCTTCAGGTGGGCCACGGTCT |
| Human *DDX58* | AGGGAGGAAGAGGTGCAGTATA | GATATCGGTTGGGATAATTCTGG |
| Human *CXCL10* | CAAACTGCCATTCTGATTTGCTGCC | GCTTTCAGTAAATTCTTGATGGCC |
| Human *CCL5* | CTACACCAGTGGCAAGTGCTCC | GGTTCAAGGACTCTCCATCCTAG |
| Human *ACTIN* | AAAGACCTGTACGCCAACAC | GTCATACTCCTGCTTGCTGAT |
