# Supplementary material for: SAFA facilitates chromatin opening of immune genes through interacting with anti-viral host RNAs
Source: PLoS Pathog. 2022 Jun 3;18(6):e1010599. doi: 10.1371/journal.ppat.1010599 (PMC9200321; doi:10.1371/journal.ppat.1010599)
Supplement: S3 Table — (DOCX) [file ppat.1010599.s009.docx]

S3 Table. Primers for ATAC-qRT-PCR

| Gene | Forward Primer (5’-3’) | Reverse Primer (5’-3’) |
| --- | --- | --- |
| Human *IFB1* | CTGGAACTGCTGCAGCTGCTT | GCTCTCCTGTTGTGCTTCTCCAC |
| Human *IFIT2* | AGAAATGCCAGGAAGACAGC | GGTGTGACACATTTCACATGG |
| Human *IFIT3* | GGACTGTCAGGCTGGAGGAAAT | TGTCCTGGCCACAGCATTG |
| Human *ISG15* | CTGACGTGTGTGCCTCAGGCTT | ATTGGCTGGCACAGAGCCCACCT |
| Human *DDX58* | ATCCTGGAAGGCTTGCAGGCTG | AAGTTCCTATGCAGCTCCGCCT |
| Human *CXCL10* | TGGTGCTGAGACTGGAGGTTCC | CCTTCGAGTCTGCAACATGGGAC |
| Human *IFITM1* | AAGGCAGAAGGAGGATGAGCC | CCATCTTCCTGTCCCTAGACTG |
| Human *IFITM3* | GCCAACCATCTTCCTGTCCCT | GTGAGAAGGGAACTCACAGGTG |
| Human *OASL* | GCTGCAGTGGCATGATCTTGG | ACCATTCCTCTGGAGTAGGCCT |
| Human *GAPDH* | CCACTAGGCGCTCACTGTTCT | CGCTGACCTTGAGCTCTCCTT |
| Human *α-ACTIN* | GGAATTGGCATGTCACCAGGG | GGAAGGAGGCGATGACCTGCT |
| Human *CCL5* | AGCAATGAGGATGACAGCGAGG | TACCGGCCAATGCTTGGTTGC |
