# Supplementary material for: SAFA facilitates chromatin opening of immune genes through interacting with anti-viral host RNAs
Source: PLoS Pathog. 2022 Jun 3;18(6):e1010599. doi: 10.1371/journal.ppat.1010599 (PMC9200321; doi:10.1371/journal.ppat.1010599)
Supplement: S4 Table — (DOCX) [file ppat.1010599.s010.docx]

S4 Table. Primers for ChIP-qRT-PCR

| Gene | Forward Primer (5’-3’) | Reverse Primer (5’-3’) |
| --- | --- | --- |
| Human *IFB1* | AGTCTCATTCCAGCCAGTGCT | GAGCTACAACTTGCTTGGATTCCT |
| Human *ISG15* | AGTGCAGTGAAGCGATCTCGG | CCCAGCACTTTGAGAGACCGA |
| Human *CCL2* | TCGCACTCTCGCCTCCAGCAT | AGCTGTGTGGTTGGGCTCACC |
| Human *CCL8* | ACCGAGGAGCAGAGAGGTTGAG | GACCTGAGCCTTTAGTTCTGG |
| Human *CXCL10* | CACACAAGATGGTGCCTCAGCA | GGTAGGTTTGCTGTTGCCTGC |
| Human *IFITM1* | ACTGGTATTCGGCTCTGTGAC | ACTGTAGACAGGTGTGTGGGT |
| Human *IFITM2* | CGATGGTAGACACAGCCATTG | CTGCTGTAGCCTTTCACCTCA |
| Human *OAS1* | ACAGTTCTGTGACTGTCCCTGC | CCACCATTACAGCACCATGCAG |
| Human *GAPDH* | AGGCTGGATGGAATGAAAGGC | AGCCCTGTAGCCTGGACCTGAT |
| Human *α-ACTIN* | AGACCACTGCAACACTGAGCA | CCTGTGCAACATAGTGAGACC |
